# Development of novel reagents to chicken FLT3, XCR1 and CSF2R for the identification and characterization of avian conventional dendritic cells

**DOI:** 10.1111/imm.13426

**Published:** 2021-11-30

**Authors:** Zhiguang Wu, Tuanjun Hu, Cosmin Chintoan‐Uta, Joni Macdonald, Mark P. Stevens, Helen Sang, David A. Hume, Pete Kaiser, Adam Balic

**Affiliations:** ^1^ The Roslin Institute University of Edinburgh, Easter Bush Midlothian UK; ^2^ Translational Research Institute Mater Research Institute‐University of Queensland Woolloongabba Qld Australia

**Keywords:** avian, chicken, conventional dendritic cell, CSF1R, FLT3, macrophage, reagents, XCR1

## Abstract

Conventional dendritic cells (cDC) are bone marrow‐derived immune cells that play a central role in linking innate and adaptive immunity. cDCs efficiently uptake, process and present antigen to naïve T cells, driving clonal expansion of antigen‐specific T‐cell responses. In chicken, vital reagents are lacking for the efficient and precise identification of cDCs. In this study, we have developed several novel reagents for the identification and characterization of chicken cDCs. Chicken *FLT3* cDNA was cloned and a monoclonal antibody to cell surface FLT3 was generated. This antibody identified a distinct FLT3^HI^ splenic subset which lack expression of signature markers for B cells, T cells or monocyte/macrophages. By combining anti‐FLT3 and *CSF1R*‐eGFP transgenic expression, three major populations within the mononuclear phagocyte system were identified in the spleen. The cDC1 subset of mammalian cDCs express the chemokine receptor XCR1. To characterize chicken cDCs, a synthetic chicken chemokine (C motif) ligand (XCL1) peptide conjugated to Alexa Fluor 647 was developed (XCL1^AF647^). Flow cytometry staining of XCL1^AF647^ on splenocytes showed that all chicken FLT3^HI^ cells exclusively express XCR1, supporting the hypothesis that this population comprises *bona fide* chicken cDCs. Further analysis revealed that chicken cDCs expressed CSF1R but lacked the expression of CSF2R. Collectively, the cell surface phenotypes of chicken cDCs were partially conserved with mammalian XCR1^+^ cDC1, with distinct differences in CSF1R and CSF2R expression compared with mammalian orthologues. These original reagents allow the efficient identification of chicken cDCs to investigate their important roles in the chicken immunity and diseases.

## Graphical abstract

We have developed tools to specifically identify chicken conventional dendritic cells (cDCs). Chicken cDCs are express high levels of FLT3 and XCR1. While the resemble the mammalian cDC1 subset, significant differences exist.

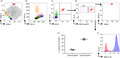

**Abbreviations**
cDC: conventional dendritic cell
CSF1R: colony‐stimulating factor 1 receptor
CSF2R: colony‐stimulating factor 2 receptor
DC: dendritic cell
FLT3: fms‐like tyrosine‐kinase 3
MHC: major histocompatibility complex
PCR: polymerase chain reaction
XCR1: X‐C motif chemokine receptor 1

## INTRODUCTION

T cell‐mediated immunity in birds, as in mammals, requires antigen uptake and presentation. While immune cell types including macrophages, monocytes and B cells can act as antigen‐presenting cells (APCs), cDCs are thought to have a central role in the maintenance of tolerance and induction of immune responses against pathogens due to their capacity to initiate primary immune responses by driving the proliferation of naïve T cells [1, 2, 3]. Mammalian cDC development largely occurs in the bone marrow (BM) and involves a developmental cascade of BM‐resident haematopoietic stem cell‐derived precursor and progenitor cells [4, 5]. In the steady state, mammalian cDC populations in peripheral tissues are maintained by pre‐cDCs or cDCs entering tissues from the blood and dividing locally [6]. In mammals, the prevailing paradigm in cDC biology is that after encountering antigen and being activated in peripheral tissues, they migrate via lymphatic vessels to draining lymph nodes where they initiate T‐cell‐dependent immune responses [4, 7]. Birds do not have specialized lymph nodes: avian secondary lymphoid tissues largely consist of poorly characterized dispersed lymphoid follicles in mucosal tissues and the skin, as well as avian‐specific mucosal lymphoid organs, such as the caecal tonsils [8, 9, 10]. In contrast to the mammalian lymph node based local immune responses, in birds it is hypothesized that antigen presentation occurs locally within tissues [11]. The precise nature and mechanisms of this antigen presentation are currently unknown.

The development of the cDC lineage (cDCpoiesis) in mammals is controlled by the growth factor Fms‐related tyrosine‐kinase 3 ligand (Flt3L) and its cognate receptor FLT3 [12]. Mammalian cDCs consist of two subsets: cDC1 and cDC2 [13]. Each cDC subset exhibits functional specialization, which is further influenced by tissue microenvironment [12, 14, 15, 16, 17]. The cDC1 subset excel at cross‐presentation of exogenous microbial and tumour antigens to efficiently prime CD8^+^ T cells and activate CD4^+^ T cells through MHC class II antigen (MHCII) presentation resulting in the polarization of activated CD4^+^ T cells towards a Th1 phenotype [18, 19, 20, 21]. The mammalian cDC2 subset exhibits less functional specialization, promoting a wide range of immune responses [22, 23, 24]. The literature is somewhat confused by the use of the term ‘DC’ to describe APCs that can be generated by cultivation of monocytes or bone marrow cells in CSF2, although monocytes are generally regarded as a separate lineage from cDC [13, 25].

Using transcriptomic approaches, a chicken immune cell population expressing genes associated with the mammalian cDC1 subset (including *XCR1*, *FLT3*, *CIITA*, *ZBTB46*, *ID2*, *IRF8*, *CADM1*) has been identified in the spleen, liver and lungs [26, 27, 28, 29]. However, due to the limited availability of avian reagents compared with mammalian species, it is unclear whether chickens have cell population equivalent to the mammalian cDC2 subsets, nor whether avian‐specific dendritic cell subsets exist. As birds lack lymph nodes, it is not clear whether chicken cDCpoiesis proceeds in the same manner as observed in mammals, nor how chicken cDCs perform the same basic functions of immune surveillance and antigen presentation as their mammalian counterparts in a radically different lymphoid tissue environment. Addressing these outstanding questions will require the production of immunological tools to identify and characterize chicken cDCs.

To address this resource gap, we previously generated antibodies against chicken CSF1 [30] and CSF1R [31] and demonstrated that labelled recombinant chicken CSF1 could bind to CSF1R‐positive cells [30]. In the current study, we describe the production and characterization of a novel anti‐chicken FLT3 monoclonal antibody and show its applications in the flow cytometric analysis and immunofluorescent staining of chicken tissue cDCs. Furthermore, we also generated reagents to label the cell surface receptors XCR1 and CSF2R, which, in combination with the anti‐chicken FLT3 monoclonal antibody, allowed us to efficiently phenotypically characterize the chicken cDC. We show that the chicken spleen contains several FLT3^+^ cell populations, with the XCR1^+^ FLT3^HI^ subset likely representing the *bona fide* chicken cDC population. In combination with a previously generated *CSF1R*‐transgenic chicken line [10], a more detailed flow cytometry analysis revealed that the cell surface phenotypes of chicken XCR1^+^ cDC were partially conserved with the mammalian XCR1^+^ cDC1 subset, with distinct differences in CSF1R and CSF2R expression compared with mammalian orthologues. We show that while *Salmonella* Typhimurium invades chicken FLT3^HI^ cDCs, they exhibit preferentially trophism for splenic macrophages. Finally, we show that the *in vitro* bone marrow‐derived DCs (BMDCs) expresses the classical chicken monocyte/macrophage marker MRC1L‐B and do not express either FLT3 or XCR1, indicating that these are more accurately described as antigen‐presenting macrophages rather than DC.

## MATERIALS AND METHODS

### Chickens and ethics statement

All birds were obtained from the National Avian Research Facility at The Roslin Institute, University of Edinburgh. All birds were hatched and housed in premises licensed under a UK Home Office Establishment License in full compliance with the Animals (Scientific Procedures) Act 1986 and the Code of Practice for Housing and Care of Animals Bred, Supplied or Used for Scientific Purposes. Production of the *CSF1R*‐eGFP reporter transgenic line has been previously described [10]. This transgenic line has been used for various studies of mononuclear phagocyte development [28, 32, 33, 34]. Breeding of transgenic chickens was carried out under the authority of Project License PPL 70/8940 with the consent of The Roslin Institute Animal Welfare and Ethical Review Board. Animals were humanely culled in accordance with Schedule 1 of the Animals (Scientific Procedures) Act 1986.

### Amplification of the full‐length chicken FLT3 gene and a short isoform

To obtain accurate sequence data for the 5’end of *FLT3*, it was firstly cloned by a mismatch RT‐PCR. Briefly, total RNA was extracted from splenocytes from a 6‐week‐old J line bird using TRIzol reagent (Invitrogen, Thermo Fisher Scientific), mRNA was then purified with Dynabeads Oligo (dT)_25_ (Invitrogen, Thermo Fisher Scientific), as per manufacturer's instruction. 2 µg mRNA was then used to create a double‐strand cDNA library using Marathon^®^ cDNA Amplification Kit (Clontech Laboratories), as per manufacturer's instructions. The mismatched adaptors were prepared as following: a forward primer 5’‐end phosphorylated AdaptL (5’ PO_4_‐TCGAGGGAA ***T*** CTTGATATCGAATTCCTGC‐3’) and a reverse primer AdaptMS (5’‐GCAGGAATTCGATATCAAG ***C*** TTCCCTCGA −3’), where bold and italic letters indicated mismatched residues. These were synthesized by Sigma‐Aldrich. 10 µl of both primers at concentration of 100 µM was mixed thoroughly, heated at 95ºC for 10 min in a water bath to anneal primers and cooled down gradually to form mismatched double‐strand adaptors. This adaptor (200 pmol) was then ligated to the above double‐strand cDNA using T4 ligase (Invitrogen). After dilution by 1 in 10 with H_2_O, the cDNA was used as PCR template to clone cDNA of 5’ end of *FLT3*, using forward primer, AP1 (5’‐GCAGGAATTCGATATCAAGA‐3’), complementary to AdaptL and reverse primer *FLT3*‐JHR3 (5’‐CTGATGACTGAGAAAGTGTA‐3’), specific to chicken *FLT3*, with the following cycling conditions: 94ºC for 1 min, 3 cycles of 53 ºC for 3 min, 3 cycles of 94ºC for 1 min, 56 ºC for 1 min and 72 ºC for 1·5 min, and 30 cycles of 94ºC for 1 min, 58 ºC for 1 min and 72 ºC for 1·5 min. Based on the DNA sequence of the *FLT3* 5’ end amplicon, a forward primer Flt3F1 (5’ATCACCAGCATGGCAGTGTGTCT3’) was synthesized, together with a reverse primer Flt3R1 (5’‐GATAACATCTTCTTAGTGGTGATGTGAA‐3’) to clone full‐length chicken *FLT3* cDNA from a splenic cDNA template, reversely transcribed from a 6‐week‐old J‐line bird and following cycling conditions: 94ºC for 3 min, 33 cycles of 94ºC 1 min 60ºC 1 min, 72ºC 3 min and then 72ºC 10 min, resulting in two amplicons, a full‐length *FLT3* (*chFLT3*) and a short isoform (*chFLT3s*).

### Semi‐quantitative RT‐PCR analysis of FLT3 expression

Tissues (thymus, spleen, bursa of Fabricius, Harderian gland, caecal tonsil, Meckel's diverticulum, bone marrow, brain, breast muscle, heart, liver, kidney, lung, small intestine and testis) were dissected from 6‐week‐old J‐line chickens. Total RNA was prepared from tissues using RNeasy mini kit (QIAGEN), following the manufacturer's instructions. 2 µg total RNA was then reverse‐transcribed into cDNA, using Superscript III (Invitrogen) as per manufacturer's instructions. Primers Flt3‐iosFwd 5’‐GCTCATCCAAATCAAACTGCT‐3’) and Flt3‐iosRev 5’‐CAACTGACATGT TAATGAAGCTC‐3’) were used for PCRs to analyse *FLT3* expression via following cycling conditions: 94ºC for 3 min, 33 cycles of 94ºC 1 min 55ºC 30 sec, 72ºC 1 min. Chicken GAPDH was used as a control gene, as described before [35].

### Expression and purification of recombinant chFLT3‐Fc and recombinant chFLT3‐V5HIS6 protein

The extracellular domains of *chFLT3* or *chFLT3*s were sub‐cloned into pKW06 to produce a fusion protein with a C‐terminal human IgG1 Fc tag or pKW08 to produce a fusion protein with a V5HIS6 tag [36]. The constructs were named as pKW06/*chFLT3*, pKW06/*chFLT3s*, pKW08/*chFLT3* and pKW08/*chFLT3s*. All constructs have their own signal peptides and were expressed in human embryonic kidney HEK293 cells as described elsewhere [37]. FLT3‐Fc was purified using a HiTrap Protein G affinity column (GE Healthcare Life Sciences). The purity and identity of FLT3‐Fc were confirmed by SDS‐PAGE and analysis of tryptic peptides by mass spectrometry (Dr Dominic Kurian, Proteomics and Metabolomics, The Roslin Institute) before immunization.

### Monoclonal antibody production, isotyping, purification and labelling

Mice were immunized with purified chFLT3‐Fc fusion protein. Immunization and cell fusion were carried out by Dundee Cell Products (DCP, Dundee, UK). Following fusion, polyclonal hybridoma cultures were tested with recombinant chFLT3‐V5HIS6 by dot‐blot and six positive hybridoma cultures were selected by DCP. These samples were then further screened by ELISA with chFLT3‐Fc, chFLT3‐V5HIS6, Fc or V5HIS6 controls and screened with *chFLT3* transfected Ba/F3 cells or control Ba/F3 cells (below). Positive samples were selected for further cloning to produce monoclonal hybridomas. Specificity of supernatant from monoclonal hybridoma cultures was again confirmed by ELISA and flow cytometry. Isotypes of these monoclonal antibodies were tested to common mouse antibody isotypes (IgG1, IgG2a, IgG2b, IgG3, IgM and IgA) and to the κ and λ light chains using the IsoStrip Mouse Monoclonal Antibody Isotyping Kit (Roche). Monoclonal hybridomas were cultured in Dulbecco's modified Eagle's medium (DMEM) with 10% Ig‐depleted foetal bovine serum (FBS). Monoclonal antibodies were purified using HiTrap Protein G affinity columns (GE Healthcare Life Sciences) and dialysed against phosphate‐buffered saline (PBS) using 30 kDa molecular weight cut‐off (MWCO) Slide‐A‐Lyser cassettes (Pierce, Thermo Fisher Scientific). The concentrations of mAbs were determined by absorbance at 280 nm with a Nanodrop. Purified mAbs were labelled with Alexa Fluor™ 647 Antibody Labelling Kit (Invitrogen). All procedures were performed according to the manufacturer's instructions.

### Transfection of Ba/F3 cell with full‐length FLT3 for screening of hybridomas

The full length of *chFLT3* or *chFLT3*s open reading frame (without stop codons) was cloned into the pEF6/V5‐His TOPO expression vector (Thermo Fisher Scientific) in‐frame with the V5 and His tags at the 3’ end of the protein. The pEF6‐*FLT3* or *FLT3s* expression constructs and pEF empty vector was transfected into Ba/F3 cells by electroporation and stably transfected Ba/F3 cells were selected with blasticidin as previously described for the chicken CSF1R gene [33]. Briefly, logarithmically growing Ba/F3 cells were harvested and resuspended in complete media at a concentration of 5 × 10^6^ cells per 200 µl. Cells were then placed in 0·4‐cm electroporation cuvettes with 50 μl of PBS containing 5 μg plasmid DNA. The cuvettes were left to equilibrate at room temperature (RT) for 10 min before electroporation at 975 μF and 300 V using a Bio‐Rad Gene Pulser electroporation apparatus. After electroporation, cells were washed in complete medium to remove surplus DNA, plated out in 6‐well plates and then incubated at 37 ºC for 24 h before adding 40 µg/ml blasticidin (Fisher Bioreagents, Fisher Scientific) for three weeks. Stable clones were selected for their survival in blasticidin.

### Screening of hybridomas and confirmation of specificity by ELISA

Supernatant from hybridoma culture was screened by indirect ELISA as described previously [38]. Briefly, assay plates (Nunc Immuno MaxiSorp, Thermo Electron LED) were coated with recombinant FLT3‐Fc, FLT3s‐Fc, FLT3‐V5H6, FLT3s‐V5H6 or relative control protein in carbonate/bicarbonate buffer (15 mM sodium carbonate, 35 mM sodium bicarbonate and 3 mM sodium azide; pH 9·6) and incubated overnight at 4 ºC. Plates were washed in PBS containing 0·1% (v/v) Tween 20 (PBS‐T) and blocked with 0·5% (w/v) casein/PBS at RT for 1 h. Neat supernatant of hybridoma culture or purified mAb was added to the plate and incubated at RT for 1 h. After three washes with PBS‐T, plates were incubated with goat anti‐mouse IgG‐horseradish peroxidase (HRP) at RT for 1 h. After a further three washes, plates were visualized by 3,3’,5,5'‐tetramethylbenzidine (TMB) substrate (Invitrogen, Thermo Fisher Scientific), and the reaction was stopped by 2N H_2_SO_4_. Plates were read at 450 nm in a SpectraMax 250 microplate spectrophotometer system (Molecular Devices, Sunnyvale, CA, USA).

### Screening of hybridomas and confirmation of specificity with FLT3‐Ba/F3 cells

Parental Ba/F3 cells, stably transfected *FLT3*‐Ba/F3, *FLT3s*‐Ba/F3 or pEF‐Ba/F3 were cultured in presence of IL‐3 (X63 medium). Cells were fixed with 1% (w/v) paraformaldehyde in PBS, permeabilized with 0·1% saponin and stained with mouse anti‐V5‐Tag antibody (Bio‐Rad) or IgG2a isotype control before adding goat anti‐mouse IgG‐fluorescein isothiocyanate (FITC; Bio‐Rad) for flow cytometric analysis. To detect surface FLT3, cells were incubated with supernatant from hybridoma culture or purified mAbs on ice for 30 min before adding goat anti‐mouse IgG (H + L) cross‐adsorbed secondary antibody, conjugated with Alexa Fluor 647 (Invitrogen, Thermo Fisher Scientific) to detect hybridoma supernatant and then analysed by flow cytometric analysis.

### Development of specific reagent to XCR1

The protein sequence of chicken XCL1 was obtained from NCBI (Accession NP_990377). The mature peptide of chicken XCL1 (previously known as lymphotactin), with the addition of a terminal lysine residue for coupling, was synthesized (Sequence: SVASQSMRKLSCVNLSTQKVDIRSIVNYEKQKVPVEAVMFITANGIRICVHPEQKWVQSAMKRIDRRRTTRRRK) and coupled with Alexa Fluor 647 (Linker‐Cys‐AF647; ALMAC Science (Edinburgh, Scotland)), yielding reagent XCL1^AF647^. The cDNA sequence for chicken *XCR1* was obtained from NCBI (Accession NM_001045838). To analyse the specificity of the reagent, the full length of *XCR1* open reading frame (without stop codon) was cloned into the pEF6/V5‐His TOPO expression vector (Thermo Fisher Scientific) in‐frame with the V5 and His tags at the 3’ end of the protein. The pEF‐*XCR1* plasmids were transfected into Ba/F3 cells and selected with blasticidin to produce stably transfected Ba/F3 (*XCR1*‐Ba/F3) as described above for the transfection of Ba/F3 cells with full‐length FLT3 for screening of hybridomas. *XCR1*‐Ba/F3 or pEF‐Ba/F3 transfected cells were then harvested and stained with XCL1^AF647^ (0·2 μg/ml) on ice for 30 min. Human IgG1‐Fc was conjugated to Alexa Fluor 647 using the AF647 Microscale Protein labelling kit (Thermo Fisher Scientific) according to manufacturer's instructions to use as peptide control. Cells were then washed twice with FACS buffer (PBS, 1·0% (w/v) bovine serum albumin and 0·05% (w/v/) sodium azide) and resuspended in FACS buffer with SYTOX^®^ Blue (Invitrogen; 1 mM solution in dimethylsulphoxide; 1/4000 dilution) immediately prior to flow cytometric analysis using a LSRFortessa (Becton Dickinson). Resulting data were analysed using FlowJo V10 software (FlowJo, Ashlan, OR. USA).

### Development of labelled CSF2 to detect CSF2R expression

Chicken CSF2 (GM‐CSF) cDNA [39] was sub‐cloned into the vector pKW06 to produce recombinant protein with a C‐terminal human IgG1 Fc tag (CSF2‐Fc) as described previously [36]. CSF2‐Fc specifically binds to chicken CSF2R on the surface of chicken granulocytes and monocytes and macrophages (Wu et al., manuscript in preparation). CSF2‐Fc was conjugated to Alexa Fluor 647 using the AF647 Microscale Protein labelling kit (Molecular Probes, Thermo Fisher Scientific) according to the manufacturer's instructions. CSF2‐Fc was also recognized with goat F(ab’) anti‐human IgG‐phycoerythrin (PE) (mouse absorbed, Cambridge BioScience) as in Table 1 when co‐staining with XCL1^AF647^.

**TABLE 1:** List of reagent used for flow cytometry and immunofluorescence staining

Reagent name/Clone	Target	Conjugate	Isotype	Source/Reference	Conc µg/ml
Mouse anti‐CD45/ clone LT−40	CD45	RPE	Mouse IgM	Southern Biotech	0·8
Mouse anti‐Bu−1/ clone AV20	Bu−1a/b	RPE	Mouse IgG1	Southern Biotech	0·1
Mouse anti‐CD3/ clone CT−3	CD3	RPE	Mouse IgG1	Southern Biotech	0·1
Mouse anti‐chicken Monocytes/Macrophages /clone KUL01	MMR1L4/MRC1L‐B	RPE	Mouse IgG1	Southern Biotech	0·4
Mouse anti‐chicken MHC II / clone 2G11	MHC class II β‐chain	RPE	Mouse IgG1	Southern Biotech	0·1
XCL1	XCR1	AF647		ALMAC	0·2
CSF2‐Fc	CSF2R	AF647	Human IgG1‐Fc	In house (Manuscript in preparation)	0·1
Mouse IgM isotype control		RPE	Mouse IgM	AlphaDiagnostic	0·8
Mouse IgG1 isotype control		RPE	Mouse IgG1	AlphaDiagnostic	0·1
Bovine IgG1‐Fc		AF647	Bovine IgG1‐Fc	In house	0·1
Goat anti‐Chicken IgY (H + L) Alexa Fluor 488		AF488	Polyclonal	Thermo Fisher Scientific	1/500

Reagent name	Target	Isotype	Supplier	Conc µg/ml	Secondary antibody
Mouse anti‐chicken FLT3	FLT3	Mouse IgG1	In house(ROS‐AV184)	0·1	Goat anti‐mouse IgG1‐PE Thermo Fisher Scientific P21129;1/2000
Mouse anti‐TIM4/clone JH9	TIM4	Mouse IgG1	In house (Hu et al., 2016)	0·1	
Mouse anti‐chicken CSF1R	CSF1R	Mouse IgG1	In house (ROS‐AV170, Garcia‐Morales, 2014)		
Mouse IgG1 isotype control/GR13·1		Mouse IgG1	In house (Hu et al., 2016)	0·1	
CSF2‐Fc	CSF2R	Human IgG1‐Fc	In house	0·1	Goat F(ab’)2 Anti‐Human IgG, Mouse ads‐PE; Cambridge BioScience 2043–09; 1/1000
Bovine IgG1‐Fc		Bovine IgG1‐Fc	In house	0·1	

Chicken splenic white blood cells (splenocytes) were isolated from *CSF1R*‐eGFP reporter transgenic birds aged from 3–20 weeks and age‐matched Hy‐line wild‐type controls. Briefly, the spleen was removed, cut into small pieces and homogenized using a borosilicate glass homogenizer (Fisher Scientific UK Ltd) with ice‐cold dissection medium (calcium‐ and magnesium‐free Hank's balanced salt solution (HBSS) with 10mM HEPES 0·45% (w/v) glucose and 1mM sodium pyruvate) and filtered through a 100‐µm cell strainer into a 50‐ml tube. Homogenized solution for birds older than 3 weeks of age was overlaid on Histopaque‐1·077 (Sigma‐Aldrich) and spun at 400 g for 20 min at RT with the brake off. Cells at the density interface were collected, washed, resuspended in cold FACS buffer and placed on ice for 10 min. Cold cells were then stained with a combination of mAbs as in Table 1 in FACS buffer for 30 min on ice in the dark. Cells were then washed three times, resuspended in cold FACS buffer and stained with SYTOX^®^ Blue Dead Cell Stain (Invitrogen; 1·0 mM stock, 1/4000 dilution) for live cell gating. Data were collected with a LRSFortessa (BD Biosciences) and analysed using FlowJo V10 software. At least 100,000 events were acquired. Dead cells were excluded by SYTOX^®^ Blue staining and doublets were then discriminated based on signal processing (FSC‐A/H). Fluorescence minus one controls (FMO) were used to confirm gating strategies. The dimensionality reduction algorithm, t‐distributed stochastic neighbour embedding (t‐SNE) was used to generate cell clusters.

### Culture of chicken BM‐derived dendritic cells

Chicken bone marrow cells were isolated from two‐week‐old *CSF1R*‐eGFP transgenic chickens (n = 3) and cultured with CSF2 and IL‐4 for 7 days as described previously [40]. Chicken BMDCs were collected by pipetting, washed with cold FACS buffer and stained with XCL1^AF647^, anti‐FLT3 and KUL01‐RPE for 30 min in FACS buffer on ice. Goat anti‐mouse IgG1 was used as secondary antibody to detect anti‐FLT3. All staining was carried out on ice and washes performed with ice‐cold buffer. Data were collected with a LRSFortessa (BD Biosciences) and analysed using FlowJo V10 software.

### In vitro staining of splenic dendritic cells and confocal imaging

Splenocytes were prepared as described above from 12‐week‐old *CSF1R*‐eGFP reporter transgenic birds. 0·2 × 10^6^ cells were plated on fibronectin‐coated 8‐well Nunc Lab‐Tek II Chamber slides (Thermo Fisher Scientific) and incubated at 41˚C with RPMI medium supplemented with 10% (v/v) heat‐inactivated FBS, 2 mM L‐glutamine and antibiotics (100 g/ml penicillin, 100 g/ml streptomycin) for one hour. After one‐hour, non‐adherent cells were gently washed off with RPMI and remaining adherent cells were incubated overnight in complete RPMI medium supplemented with 200 ng/ml recombinant chicken CSF1 produced as described previously [30]. The next day slides were chilled on ice for 30 minutes and stained using XCL1^AF647^, anti‐chicken MHCII‐RPE and KUL01‐RPE (Table 1) in RPMI. After one hour, cells were washed in phenol red‐free RMPI medium. After staining, phenol red‐free RMPI with 10% (v/v) FBS was added to the cells and the chamber slides were placed on a microscope stage heated to 41˚C and imaged using a Zeiss LSM 710‐inverted microscope.

### Immunofluorescent staining of FLT3^+^ cells and confocal imaging of tissue sections

Unfixed tissue sections from four‐week‐old birds were embedded in Cellpath™ OCT Embedding Matrix (Fisher Scientific UK Ltd, Loughborough, UK) and snap‐frozen at −80˚C for two hours. 10‐µm sections were cut onto Superfrost Plus slides (Menzel‐Gläser, Braunschweig, Germany), air‐dried for one hour at room temperature before being fixed with 100% methanol at 4°C for 10 min and then air‐dried for a further one hour at room temperature. All primary antibodies used in this study are shown in Table 1. All slides were blocked for one hour in 2·5% skimmed milk powder (Oxoid Ltd., Basingstoke, UK), 2·5% normal horse serum (Sigma, Gillingham, UK), 0·1% Triton X‐100 (Sigma, Gillingham, UK) in PBS (MST‐PBS). Isotype‐matched antibody controls (Table 1) were added at the same concentration as primary antibodies. Primary antibodies: goat anti‐chicken IgY (H + L), Alexa Fluor 488 (Thermo Fisher Scientific (Life Technologies Ltd.), Renfrew, UK) used at 1/500 dilution; all other antibodies used at 1/100 dilution. All primary antibodies diluted in blocking reagent (above) and incubated at 4°C overnight, washed for 20 minutes in PBS, followed by incubation with secondary antibodies for two hours (donkey anti‐mouse IgG Alexa Fluor 594, donkey anti‐mouse IgG1 Alexa Fluor 594, donkey anti‐mouse IgG2a Alexa Fluor 647; Thermo Fisher Scientific (Life Technologies Ltd.)) used at 1/300 dilution and mounted in ProLong^®^ Gold Antifade Mountant (Thermo Fisher Scientific (Life Technologies Ltd.)). Where appropriate, sections were counterstained with 1 μg/ml 4′, 6′‐diamidino‐2‐phenylindole (DAPI; Sigma, Gillingham, UK) in the final incubation step. Samples were imaged using an inverted confocal microscope (Zeiss LSM710) and images analyses using Zeiss ZEN 3·1 software.

### Preparation of bacteria and phagocytosis assay

*Salmonella enterica* serovar Typhimurium strain ST4/74 nal^R^ and an isogenic mutant lacking the function of type III secretion system 1(Δ*prgH*) which promotes bacterial invasion were used in this study [32]. Both ST4/74 nal^R^ and ST4/74 nal^R^ Δ*prgH* were engineered to constitutively express mCherry by transformation with a derivative of pFVP25·1 [41] (*Salmonella*‐mCherry). Bacteria were streaked from frozen glycerol stocks onto fresh Luria Bertani (LB) agar plates and incubated at 37°C overnight. A single bacterial colony was inoculated into LB broth and incubated in a shaking incubator overnight at 37°C. Optical density at 600 nm (O.D.600) of the overnight culture was measured to determine the bacterial count, and the culture was diluted in RPMI 1640 (Gibco, Thermo Fisher Scientific) with 10% (v/v) FBS to achieve the desired multiplicity of infection (MOI). The bacterial viability and the MOI were retrospectively determined by plating of serial ten‐fold culture dilutions to agar medium.

Single‐cell suspensions of splenocytes from 11‐week‐old *CSF1R*‐eGFP transgenic reporter birds [10] were isolated as described above, washed with RPMI1620 twice, resuspended in RPMI1640 with 10% (v/c) FBS and plated into a 96‐well U‐bottom plate. The above two stains of *Salmonella*‐mCherry were added at a MOI of 5 for 45 min at 41 °C. A duplicated plate with cells was pre‐chilled, and the same number of bacteria was added and left on ice for controls. Cells were then washed and incubated with RPMI with 10% (v/v) FBS containing 100 μg/ml gentamicin (Thermo Fisher Scientific) for 30 min to kill extracellular *Salmonella*. Then, the cells were washed with cold PBS, stained with FLT3 mAb and analysed by flow cytometry as described above. Two independent phagocytosis assays were performed, and six *CSF1R*‐eGFP birds and two Hy‐Line wild‐type birds (flow control) were used in total. Results are presented as the relative percentage of mCherry‐expressing cells in each population (the mean ±standard deviation (SD)). Geometric mean fluorescence intensity (MFI) was used to measure the expression level of mCherry. The integrated mean fluorescence intensity (iMFI) [42, 43] was introduced to reflect the total functional response of mCherry^+^ cells. iMFI is computed by multiplying the relative frequency (% positive) of cells expressing mCherry with MFI of mCherry^+^ population.

Statistical analysis was conducted using a two‐tailed unpaired t‐test with Welch correction. Statistical significance was defined as follows: ∗, *p* < 0·05; ∗∗, *p* < 0·01; and ∗∗∗, *p* < 0·001. All analyses were performed using the statistical program PRISM 7 (GraphPad).

## RESULTS

### Chicken FLT3 gene and isoforms

**FIGURE 1:**
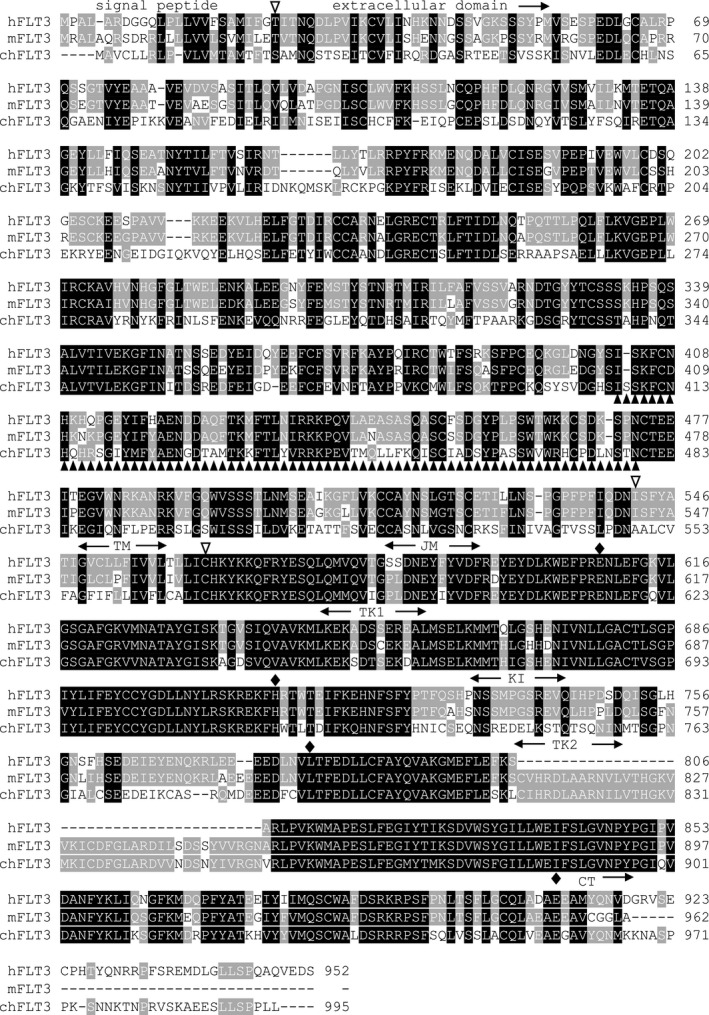
Alignment of the predicted chicken fms‐like tyrosine‐kinase 3 (chFLT3) amino acid (aa) sequence with those of human (P36888‐2) and mouse (Q3UEW6) *Flt3*, with reference to secondary structural features. Shaded areas represent conservation of amino acid similarity—the darker the shading, the more conserved the residue across species. Dashes indicate gaps in the alignment. Empty reverse triangles above the sequences indicate the start points of each domain, and filled diamonds label the start points of each substructure of intracellular domain. TM: transmembrane domain, JM: juxtamembrane domain, TK1: tyrosine‐kinase 1 domain, KI: tyrosine‐kinase insert domain, TK2: tyrosine‐kinase 2 domain, CT: C terminus. Filled triangles indicate that those residues are lack in chFLT3s isoform

The only chicken FLT3 transcript annotated in ENSEMBL (ENSGALT00000080989·3) encodes an 877 amino acid predicted protein, truncated at the N terminus relative to orthologs in turkey and zebrafinch. Using 5’ rapid amplification of cDNA ends (RACE) method, we amplified an extended cDNA product of approximately 460 base pairs (bps) in length, that contained a short‐length of 5’ untranslated region, a start codon and a signal peptide region of chicken *FLT3 (chFLT3)*, which corrected the prediction in the ENSEMBL database for a signal peptide region of *chFLT3*. Based on this information, we cloned full‐length of *FLT3* cDNA. The largest open reading frame of *chFLT3* is 2,988 bps and composed of 24 coding exons. The predicted protein sequence of *chFLT3* has 50·5% and 51·6% identity with human and murine FLT3, respectively. C*hFLT3* gene encodes 995 amino acids, including a signal peptide region (aa 1–19), an extracellular domain (aa 20–548), a transmembrane domain (aa 549–569) and an intracellular domain (aa 570–995), which contains a tyrosine‐kinase domain consisting of 2 lobes joined by a tyrosine‐kinase insert and a C terminus (Figure 1). In our PCR cloning, we also identified an isoform of *chFLT3* with a deletion mutation, named as *chFLT3*s. The *chFLT3*s is 219 bps shorter than *chFLT3*, with a deletion of 107 bps in 3’ end of exon 9 and 112 bps in 5’ end of exon 10, resulting in a 73‐aa deletion located at the N terminal end of the extracellular domain in comparison with *chFLT3* (Figure 1).

### Tissue distribution and cellular expression of chicken FLT3

**FIGURE 2:**
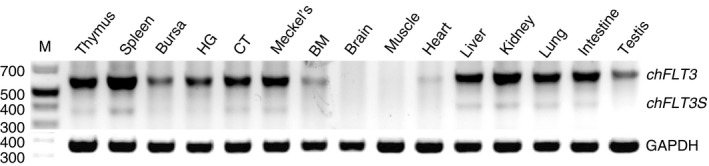
Tissue expression of *chFLT3* isoforms. Tissues were dissected from a 6‐week‐old male J line, including lymphoid and non‐lymphoid chicken tissues, where HG refers to Harderian gland; CT, caecal tonsil; Meckel's, Meckel's diverticulum; BM, bone marrow. M represents a DNA ladder (bp) and the chicken GAPDH cDNA is a control. The results shown are representative of two independent experiments

The expression patterns of *chFLT3* isoforms were analysed by RT‐PCR using Flt3‐iosFwd/iosRev primers. In contrast to rather restricted expression of human and murine *FLT3* (see www.biogps.com), *chFLT3* was widely expressed at the mRNA level in lymphoid and most non‐lymphoid tissues analysed apart from brain, muscle and heart. *ChFLT3* was a predominant isoform, whereas *chFLT3s* was only weakly expressed (Figure 2). The widespread expression is confirmed in a large‐scale meta‐analysis of gene expression data to produce a chicken gene expression atlas [44].

### Generation of mouse anti‐chFLT3 monoclonal antibodies

To generate monoclonal antibodies recognizing chicken FLT3, mice were immunized with FLT3‐Fc and hybridomas generated as described in Methods and Materials. After initial screening, six hybridoma supernatants were further screened by ELISA (Figure S1A). Three produced antibodies that bound specifically to FLT3‐Fc and FLT3‐V5H6, but not FLT3s‐Fc or FLT3s‐V5H6, Fc or V5H6 controls. To enable studies of the binding specificity of mAb to FLT3, Ba/F3 cells were stably transfected with full‐length FLT3 or FLT3s. The success of transfection was tested by intracellular staining against the V5 tag. Anti‐V5 staining (Figure S1B) showed around 36% of Ba/F3 cells were successfully transfected for *chFLT3* or *chFLT3s*. Two hybridoma supernatants (4E7 and 8A5) among the three tested were found to bind Ba/F3 cells stably transfected with full‐length *FLT3* (*FLT3*‐Ba/F3), but not the short form (*FLT3s*‐Ba/F3) or empty pEF6 vector transfected Ba/F3 cells (pEF‐Ba/F3). All three hybridomas selected by either ELISA or flow cytometry analysis were further cloned to produce monoclonal hybridomas. Unfortunately, 8A5 stopped producing antibody after sub‐cloning. 4E7 and 8D12 were isotyped and found to be IgG1. These two monoclonal hybridomas were designated as ROS‐AV184 (4E7) and ROS‐AV185 (8D12) for future reference.

### Confirmation of the specificity and utility of the anti‐chFLT3 monoclonal antibody

**FIGURE 3:**
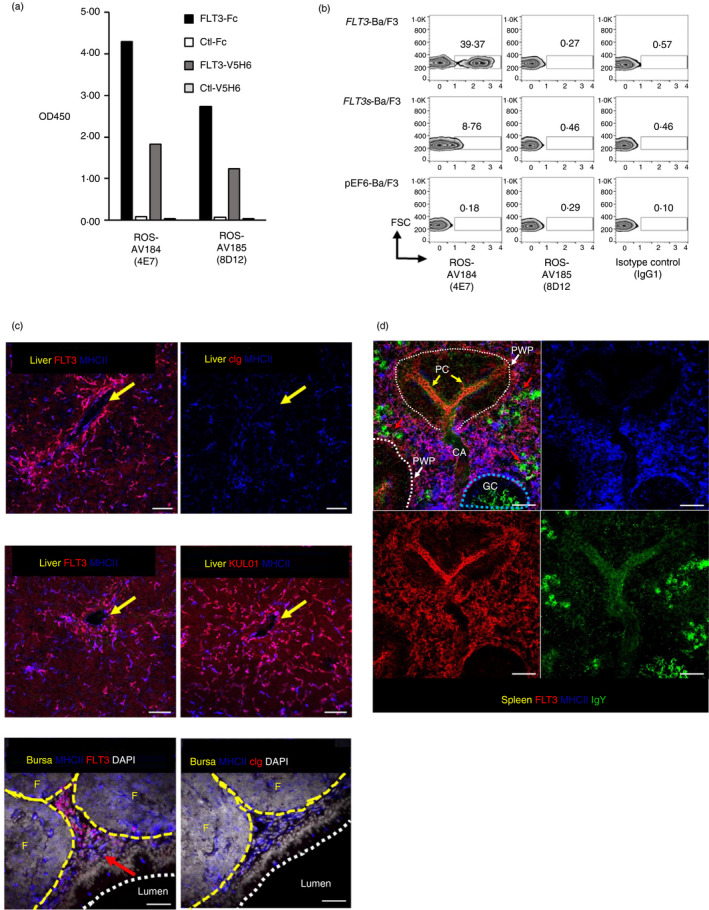
Confirmation of the specificity of purified monoclonal antibodies for FLT3 by ELISA (a) and flow cytometric analysis on transfected Ba/F3 cells (b). Plates were coated with chFLT3 or control protein. Purified antibodies ROS‐AV184 (4E7) and ROS‐AV185 (8A5) effectively recognized chFLT3 not control proteins by ELISA (a). Purified ROS‐AV184 (4E7) specially labelled *FLT3*‐Ba/F3 not *FLT3s*‐Ba/F3 or control Ba/F3 (b). Y‐axis = FSC‐H; purified mAbs or IgG1 isotype control as detected by secondary goat anti‐mouse IgG1‐AF647. (c and d). Confocal analysis of anti‐FLT3 (ROS‐AV184) staining on sections of liver, bursa and spleen. Co‐staining of anti‐FLT3 and MHC II on liver sections revealed that liver FLT3^+^ cells were scattered throughout the parenchyma and concentrated around blood vessels (yellow arrows); scale bar = 20 µm. In contrast, MRC1L‐B^+^ MHCII^+^ liver macrophages and Kupffer cells were more abundant in the liver parenchyma and not concentrated around blood vessels (yellow arrows); scale bar = 20 µm. Co‐staining of anti‐FLT3 and MHC II in the bursa of Fabricius revealed that FLT3^+^ MHCII^+^ cell were located in the interfollicular regions (red arrow), but not with B‐cell follicles (labelled as F); Scale bar =20 µm. Co‐staining of anti‐FLT3 and MHC II on the spleen sections demonstrated that FLT3^+^ MHCII^+^ cells were abundant in the red pulp but not detected in the periellipsoid white pulp (PWP), nor germinal centres (GC). Anti‐chFLT3 also stained the MHCII^−^ ellipsoid blood vessels (penicillary capillaries, PC), but not other blood vessels (including the central artery, CA); scale bar = 50 µm

Monoclonal antibodies ROS‐AV184 and ROS‐AV185 were purified and further tested by ELISA and flow cytometry analysis on Ba/F3 cells (Figure 3). Both ROS‐AV184 and ROS‐AV185 specifically recognized FLT3 by ELISA (Figure 3a). As in the screening, ROS‐AV184 recognized *FLT3*‐Ba/F3, but not *FLT3s*‐Ba/F3 or pEF‐Ba/F3 control cells. ROS‐AV185 failed to stain transfected Ba/F3 cells (Figure 3b). Henceforward, ROS‐AV184 (4E7) was used as mouse anti‐chicken FLT3 as listed in Table 1. Anti‐chFLT3 was used to detect FLT3^+^ cells *in situ* by immunofluorescence staining. Anti‐chFLT3 did not detect specific cell populations in paraformaldehyde‐fixed or acetone‐fixed tissues (data not shown). In methanol fixed sections, anti‐chFLT3 stained ramified MHCII^+^ cells in the liver (Figure 3c; Figure S2A‐D). Liver FLT3^+^ cells were scattered throughout the parenchyma and concentrated around blood vessels (Figure 3c; Figure S2A‐D). In contrast, MRC1L‐B^+^ MHCII^+^ liver macrophages and Kupffer cells [27] were more abundant in the liver parenchyma and not concentrated around blood vessels (Figure 3c; Figure S2C). In the bursa of Fabricius FLT3^+^ MHCII^+^ cell were located in the interfollicular regions, but not with B‐cell follicles (Figure 3c; Figure S2E,F). In the spleen, FLT3^+^ MHCII^+^ cells were abundant in the red pulp but not detected in the periellipsoid white pulp (PWP), nor germinal centres (GC) (Figure 3d; Figure S3A, B). Anti‐chFLT3 also stained the MHCII^−^ ellipsoid blood vessels, but not other blood vessels (including the central artery; Figure 3d; Figure S3Ci, Cii).

### Identification of splenic conventional dendritic cells using the anti‐chFLT3 monoclonal antibody ROS‐AV184

**FIGURE 4:**
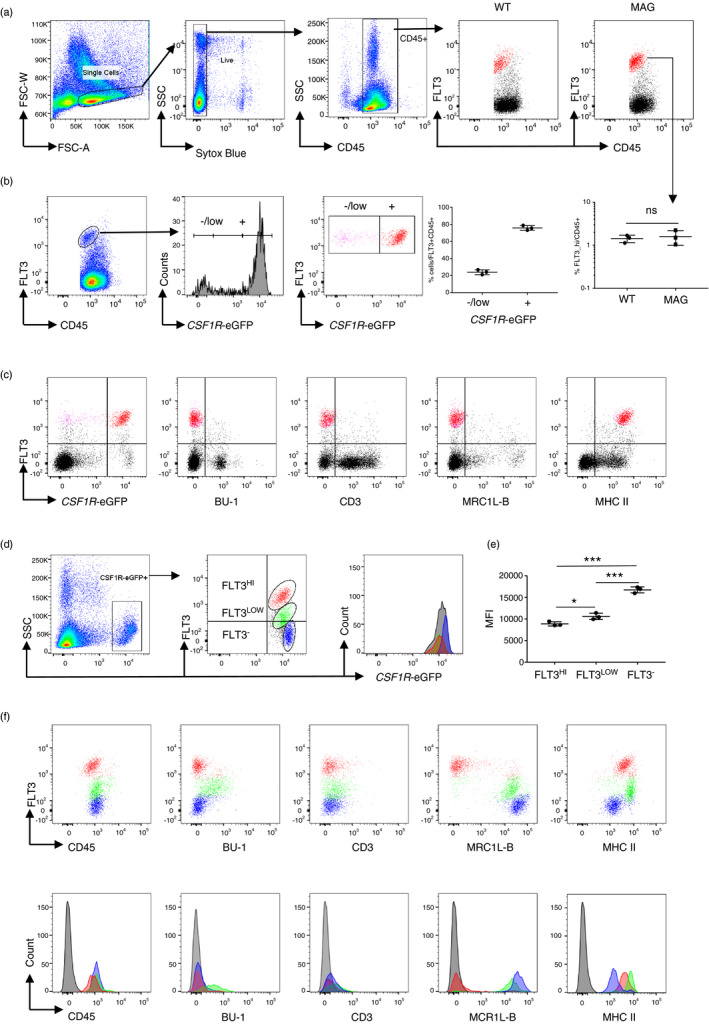
Identification of splenic cDC using the anti‐chFLT3 monoclonal antibody (ROS‐AV184) by flow cytometric analysis. (a) Identification of FLT3^HI^CD45^+^ population in splenocytes from 3 weeks old Hy‐line wild‐type (WT) or *CSF1R*‐eGFP transgenic birds. Single, live and CD45^+^ cells were gated for the analysis. FLT3^HI^CD45^+^ cells were identified and coloured in red. The relative percentages of FLT3^HI^ in CD45^+^ populations between two lines of birds were compared. (b) The statue of *CSF1R*‐eGFP expression on FLT3^HI^CD45^+^ cells. FLT3^HI^CD45^+^ cells were gated as FLT3^HI^ *CSF1R*‐eGFP^−/LOW^ and FLT3^HI^ *CSF1R*‐eGFP^+^ in histogram based on their expression levels of *CSF1R*‐eGFP and overlaid in dot plot to show both populations (FLT3^HI^ *CSF1R*‐eGFP^−/LOW^ in purple and FLT3^HI^ *CSF1R*‐eGFP^+^ in red). The percentages of FLT3^HI^ *CSF1R*‐eGFP^−/LOW^ and FLT3^HI^ *CSF1R*‐eGFP^+^ are shown on the left. (c) Phenotypes of splenic FLT3^HI^ population in terms of *CSF1R*‐eGFP, BU‐1, CD3, MRC1L‐B and MHC II as determined by co‐staining of anti‐FLT3 with anti‐BU‐1, anti‐CD3, KUL01 or anti‐MHCII. (d) *CSF1R*‐eGFP^+^ cells were gated and further separated into three populations based on their expression levels of FLT3. Black, isotype control of *CSF1R*‐eGFP^+^ cells; red, FLT3^HI^ population; green, FLT3^LOW^ population; blue, FLT3^−^ population. (e) MFI of *CSF1R*‐eGFP expression across the above three populations. (f) Expression of CD45, BU‐1, CD3, MRC1L‐B and MHCII on *CSF1R*‐eGFP^+^ FLT3^HI^, FLT3^LOW^ and FLT3^−^ populations. Top, coloured dot plot showing overlays of the three populations; bottom, histogram showing the phenotypes of the three populations. Black, isotype controls. Representative plots from one of three animals. Data were analysed using two‐tailed unpaired t‐test with Welch correction. Data are presented as mean ±SD of three birds

In mammals, FLT3 is highly expressed on haematopoietic progenitor cells and is progressively lost in cells committing to the B cell, T cell, granulocyte/macrophage and megakaryocyte/erythrocyte lineages [12, 14, 45], whereas expression is maintained in the dendritic cell lineages [46]). In RNA expression analysis, chicken cDCs but not macrophages, T cells nor B cells were found to express *FLT3^26^*. Most if not all DC in mouse also express CSF1R mRNA, albeit at the lowest levels in cDC1s, and in non‐lymphoid tissues are CSF1R‐dependent [47]. In the current study, anti‐chFLT3 was used to stain cell surface FLT3 in chicken splenocytes. Figure 4a shows that high levels of FLT3 were detected in a subpopulation of CD45^+^ cells in both wild‐type (WT) and *CSF1R*‐eGFP transgenic birds. The majority (~80%) of FLT3^HI^ CD45^+^ cells expressed the *CSF1R*‐eGFP transgene (Figure 4a,b). Staining for cell lineage markers Bu‐1(B cells), CD3 (T cells) or MRC1L‐B (macrophages) was not observed in FLT3^HI^ cells, irrespective of *CSF1R*‐transgene expression (Figure 4c). FLT3^HI^ cells expressed high levels of MHCII, consistent with immune‐fluorescence staining of tissues (Figure 3c,d). In the *CSF1R*‐eGFP^−^ population, FLT3 was expressed at low‐to‐intermediate levels, (Figure S4). *CSF1R*‐eGFP^−^ FLT3^INT^ cells did not consist of a discrete cell population in our analysis, with *CSF1R*‐eGFP^−^ FLT3^INT^ cells expressing variable levels of MHCII, MRC1L‐B, CD3 and Bu‐1 (Figure S4).

We then gated on *CSF1R*‐eGFP^+^ cells and further segregated these cells into three populations based on FLT3 expression levels (FLT3^HI^, FLT3^LOW^ and FLT3^−^ populations, Figure 4d,e,f). The *CSF1R*‐eGFP^+^ FLT3^HI^ and FLT3^LOW^ populations showed significantly lower levels of *CSF1R*‐transgene expression than the FLT3^−^ population (Figure 4e). The geometric mean fluorescent intensity (MFI) of *CSF1R*‐eGFP expression was significantly different between the FLT3^HI^ or FLT3^LOW^ populations and FLT3^−^ population (Figure 4e). The MFI of *CSF1R*‐eGFP expression of the FLT3^HI^ population was also marginally lower than the FLT3^LOW^ population (Figure 4e). The FLT3^HI^ population was CD45^+^ Bu‐1^−^ CD3^−^ MRC1L‐B^−^ MHCII^HI^. Two MRC1L‐B^+^ populations were identified. The FLT3^LOW^ population was MRC1L‐B^+^ Bu‐1^LOW^ MHCII^HI^, previously identified as a chicken splenic macrophage subpopulation [48], whereas the FLT3^−^ MRC1L‐B^+^ MHCII^+^ cells represent ‘classical’ chicken splenic macrophages [49].

### Development of specific reagent to XCR1

**FIGURE 5:**
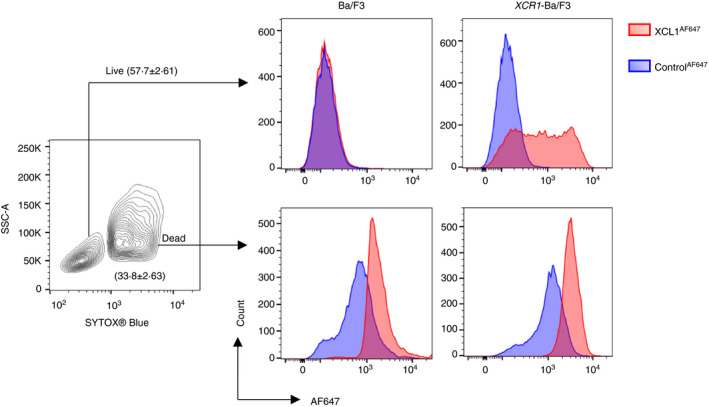
XCL^AF647^ specifically binds to *XCR1*‐Ba/F3 and dead cells. Ba/F3 cells transfected with *XCR1* or pEF control plasmids were labelled with XCL^AF647^ or control peptide^AF647^ and gated into live/dead cells based on SYTOX^®^ Blue staining profiles. XCL1^AF647^ was found to stain live *XCR1* transfected Ba/F3 not control Ba/F3 cells. XCL1^AF647^ labelled dead Ba/F3 cells regardless of *XCR1* transfection

The chemokine XCL1 is the sole ligand for XCR1, which has been reported to be a highly specific functional marker for the cDC1 subset in mammals [47, 49, 50, 51]. Transcriptional analysis of chicken MPS cells indicated that *XCR1* is co‐expressed with other genes associated with the mammalian cDC1 subset (including *FLT3*, *CIITA*, *ZBTB46*, *ID2*, *IRF8*, *CADM1*) in the spleen, liver and lungs [26, 27, 28]. Therefore, we investigated whether XCR1 had similar DC‐associated expression in chickens. The unique structure of chemokine receptors which have seven transmembrane spanning domains make production of monoclonal antibodies to XCR1 non‐trivial. As an alternative approach, receptor detection using fluorescent ligands has been developed and widely used for detection of chemokine receptors using flow cytometry [52, 53, 54, 55]. The relatively small size of XCL1 enables it to be produced by total peptide synthesis and we used this approach to generate a derivative conjugated to Alexa Fluor 647 (XCL1^AF647^). XCL1^AF647^ was first shown to bind specifically to Ba/F3 cells stably transfected with XCR1 (Figure 5). In mammals, XCR1^+^ DCs are superior to other DC subsets in internalizing dead or dying cells [56], consistent with their role in the cross‐presentation of antigen to CD8^+^ T cells [50, 55, 56, 57, 58]. Binding studies suggested that XCL1 marked stressed and dead cells for uptake into cross‐presenting DCs [59]. Similarly, chicken XCL1^AF647^ also bound to dead cells irrespective of XCR1 (Figure 5) expression, further strengthening support for a role of XCL1 in the recognition and uptake of dead cells (or their derivatives) by XCR1^+^ cDCs.

### The chemokine receptor XCR1 is selectively expressed on chicken FLT3^HI^ cDCs

**FIGURE 6:**
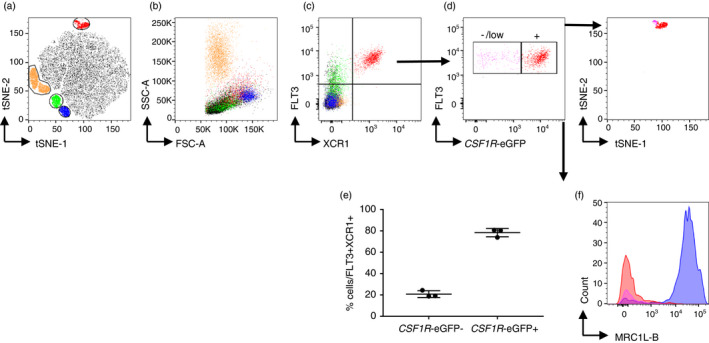
XCR1 is selectively expressed on FLT3^HI^ cDC. Splenocytes were isolated from 3‐week‐old *CSF1R*‐eGFP transgenic chickens and stained for XCR1 and FLT3. (a) tSNE visualization of all live cells. tSNE plots were generated based on the SSC‐A, FSC‐A, *CSF1R*‐eGFP, FLT3 and XCR1 staining. (b) Cell clusters were backgated on SSC/FSC plot to confirm the size and granularity of different populations and to identify heterophils. (c) Cell clusters were backgated on XCR1/FLT3 plot to confirm their identities. Yellow = heterophils; red = FLT3^HI^XCR1^+^ cDC; blue = *CSF1R*‐eGFP^+^FLT3^−^XCR1^−^ macrophages; green = *CSF1R*‐eGFP^+^FLT3^LOW^XCR1^−^ population. (d) The FLT3^HI^XCR1^+^ cDC cluster was gated into *CSF1R*‐eGFP^−/LOW^ and *CSF1R*‐eGFP^+^ populations based on their expression levels of *CSF1R*‐eGFP and backgated on to the tSNE plot. Purple = FLT3^HI^XCR1^+^ *CSF1R*‐eGFP^−^ population; red = FLT3^HI^XCR1^+^ *CSF1R*‐eGFP^+^. (e) The percentages of FLT3^HI^XCR1^+^ *CSF1R*‐eGFP^−^ population and FLT3^HI^XCR1^+^ *CSF1R*‐eGFP^+^ populations (n = 3). (f) Histogram showing the expression levels of macrophage marker MRC1L‐B on the surface of FLT3^HI^XCR1^+^ *CSF1R*‐GFP^−^ (purple) and FLT3^HI^XCR1^+^ *CSF1R*‐eGFP^+^ (red) with *CSF1R*‐eGFP^+^FLT3^−^XCR1^−^ macrophages (blue) as MRC1L‐B^+^ control

To investigate whether chicken XCR1 expression is conserved on the surface of chicken cDCs, we analysed XCR1 expression in splenic cells using the newly developed reagent XCL1^AF647^ in combination with staining for FLT3 (Figure 6). Co‐staining of FLT3 and XCR1 on live splenic cells was from *CSF1R*‐eGFP transgenic chickens were visualized using t‐SNE [60]. XCR1 staining was restricted to the FLT3^HI^ cell population (Figure 6a‐c). ~80% of FLT3^HI^ XCR1^+^ cells expressed the *CSF1R*‐eGFP transgene (Figure 6d,e). The FLT3^HI^ XCR1^+^ population, regardless of *CSF1R*‐eGFP status, did not express MRC1L‐B (Figure 6f). Based on co‐expression of FLT3 and XCR1, we designated the FLT3^HI^ XCR1^+^ subset as chicken cDCs.

### Chicken cDC have a distinct cell surface phenotype compared to mammalian orthologues

**FIGURE 7:**
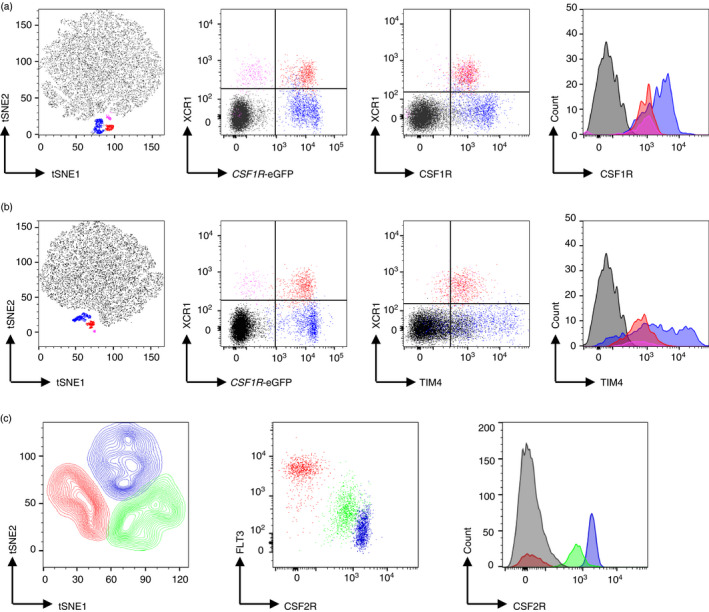
Cell surface phenotype of splenic cDC. Cell surface expression of CSF1R (a) or TIM4 (b) by splenic cDC. Splenocytes from 20‐week‐old *CSF1R*‐eGFP birds were isolated and stained with XCL1^AF647^ with anti‐CSF1R mAb or anti‐TIM4 as in Table 1. tSNE plots of all single live cells were generated based on the SSC‐A, FSC‐A, *CSF1R*‐eGFP, XCR1 and CSF1R or TIM4 staining. Separated cell clusters were backgated to identify cDC and macrophage populations. Red = *CSF1R*‐eGFP^+^XCR1^+^ cDC; purple = *CSF1R*‐eGFP^−^XCR1^+^ cDC; blue = *CSF1R*‐eGFP^+^ XCR1^−^ macrophages; black = isotype controls in histogram. (c) Cell surface expression of CSF2R by splenic cDC. Splenocytes from 3‐week‐old *CSF1R*‐eGFP^+^ birds were isolated and stained with anti‐FLT3 mAb with CSF2‐Fc. tSNE plots of *CSF1R*‐eGFP^+^ cells were generated based on the SSC‐A, FSC‐A, FLT3 and CSF2R staining. Separated cell clusters were backgated to confirm the phenotype of splenic cDC and macrophage populations. Red = *CSF1R*‐eGFP^+^FLT3^HI^CSF2R^−^ cDC; blue = *CSF1R*‐eGFP^+^FLT3^−^CSF2R^+^ macrophages; green = *CSF1R*‐eGFP^+^FLT3^LOW^CSF2R^int^ macrophages; black = isotype controls in histogram

To further characterize the cell surface phenotype of chicken cDC and macrophage populations, the expression profiles of CSF1R [31], CSF2R or TIM4 [27] were analysed (Figure 7). CSF1R‐dependent signalling controls macrophage differentiation and survival. In mammals, CSF1R is not detected [2, 61, 62, 63] or weakly detected [47] in the XCR1^+^ cDC1 subset. In contrast, chicken XCR1^+^ cDCs showed expression of the CSF1R (Figure 7a), as detected by staining with anti‐CSF1R mAb [31], albeit at lower levels compared with XCR1^−^ *CSF1R*‐eGFP^+^ macrophages (Figure 7a). There was no difference in the level of CSF1R staining between *CSF1R*‐eGFP^−^ and *CSF1R*‐eGFP^+^ cells (Figure 7a).

TIM4 is cell surface receptor for apoptotic cells [27]. XCR1^+^ cDCs expressed TIM4 at an intermediate level compared with other *CSF1R*‐eGFP^+^ populations, irrespective of *CSF1R*‐eGFP expression (Figure 7b). As in Figure 4, all three populations expressed high level of MHCII. These data suggest that all three populations are potential APCs, whereas TIM4‐dependent recognition of apoptotic cells is restricted to FLT3^HI^ cDCs and a subset of splenic macrophages [27].

Granulocyte‐macrophage colony‐stimulating factor (GM‐CSF, CSF2) is a growth factor that controls the differentiation of the haematopoietic progenitors [64]. In mammals, GM‐CSF controls terminal differentiation of XCR1^+^ cDCs [65] and non‐lymphoid tissue dendritic cell homeostasis [66]. The CSF2 receptor (CSF2R, also known as GM‐CSFR) is a heterodimer composed of the beta common chain (also shared with the receptors for IL‐3 and IL‐5) and the CSF2‐specific alpha chain (CSF2RA) [67, 68]. The chicken genome contains three *CSF2RA* paralogues, and it remains unclear in which paralogue represents the *bona fide CSF2RA* [27]. In the absence of specific antibodies to the different chicken CSF2RA paralogues, we used a strategy previously used to characterize functional CSF2R expressing cells in murine studies [69] and our own studies with chicken CSF1 [30]. A novel avian reagent for labelling the chicken CSF2R was created by fluorescently tagging a recombinant chimeric protein containing chicken CSF2 coupled to human Fc of IgG1. This CSF2‐Fc reagent labels a large number of cell types in the spleen, including granulocytes (data not shown). To better visualize MPS populations, tSNE analysis was performed on *CSF1R*‐eGFP^+^ cells (Figure 7c). Chicken cDCs (*CSF1R*‐eGFP^+^ FLT3^HI^ cells) lacked the expression of CSF2R whereas FLT3^LOW^ and FLT3^−^ macrophage subsets were clearly labelled with the CSF2‐Fc reagent (Figure 7c).

### Cultured chicken BMDCs do not phenotypically represent in vivo cDCs

**FIGURE 8:**
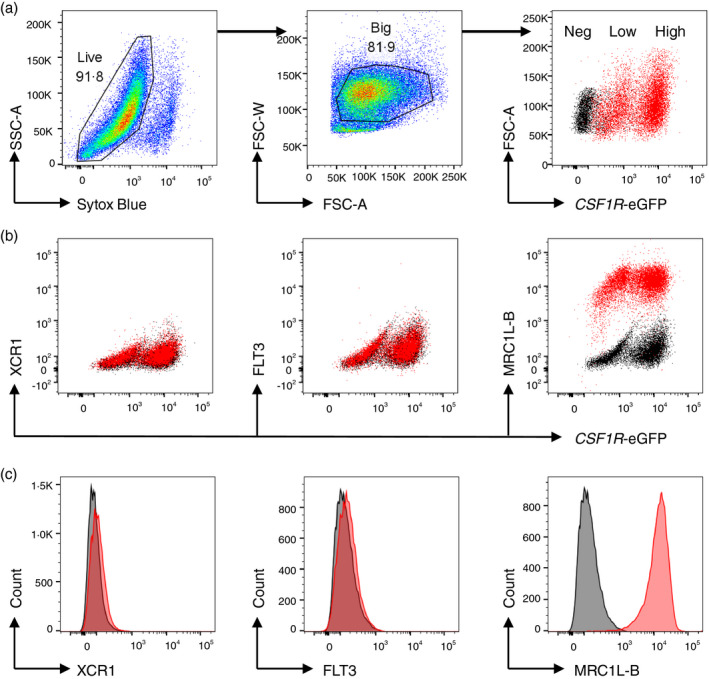
Flow cytometric analysis of surface expression of XCR1, FLT3 and MRC1L‐B (red) on BMDC. Relative FMO are shown as controls (black). Representative data from three birds. (a) Live, big and *CSF1R*‐eGFP^+^ cells (low and high as shown in red) were gated for analysis. *CSF1R*‐eGFP^LOW^ and *CSF1R*‐eGFP^HI^ populations were shown. (b) Dot plot shows that both *CSF1R*‐eGFP^HI^ and *CSF1R*‐eGFP^LOW^ cells did not express XCR1 and FLT3 but expressed MRC1L‐B. (c) Histograms for XCR1, FLT3 or MRC1L‐B staining (red) and FMO (black)

Bone marrow (BM) cells cultured in CSF2 and IL‐4 have APC activity and have been referred to as BM‐derived DC (BMDC) [70, 71]. To determine whether chicken BMDCs are representative of *in vivo* XCR1^+^ FLT3^HI^ cDCs, chicken BMDCs were cultured as previously described [40] and the cell surface phenotypes were investigated using the newly developed reagents. The cultured BMDCs were heterogeneous populations but mostly large cells with two levels of *CSF1R*‐eGFP expression (Figure 8a). Both *CSF1R*‐eGFP^HI^ and *CSF1R*‐eGFP^LOW^ populations expressed MRC1L‐B, but neither had detectable XCR1 or FLT3 (Figure 8b,c). In chickens, bone marrow‐derived macrophages grown in CSF1 also express high levels of MHCII, but did not express detectable FLT3 or XCR1 mRNA [72].

### Appearance of chicken cDCs

**FIGURE 9:**
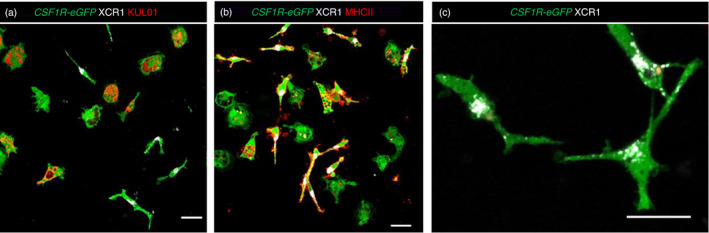
Morphology and phenotype of chicken splenic dendritic cells. Chicken splenocytes were isolated from a 12‐week‐old *CSF1R*‐eGFP^+^ transgenic birds and cultured in fibronectin‐coated chamber slides overnight and stained for XCR1and MRC1L‐B (a), XCR1 and MHCII (b) or XCR1 only (c). Scale bar = 10 µm

The term ‘dendritic cell’ was originally coined to describe an adherent cell type, isolated from murine lymphoid organs, which could be differentiated from macrophages on the basis of a tree like (i.e. ‘dendritic’) morphology and lack of endocytic function [73]. We compared the morphology of chicken cDCs and macrophages in adherent cell cultures from the spleens of *CSF1R*‐eGFP transgenic chickens to determine whether these cell types could be distinguished morphologically from each other. To avoid fixation dependent changes to cDC morphology, we performed confocal microscopic analysis of live cells. Two main cell morphologies of adherent splenic cells were observed, rounded cells with ruffled edges and many intracellular vacuoles and elongated cells with dendritic projections that lacked ruffled edges and obvious intracellular vacuoles. The former population consisted of MRC1L‐B^+^ macrophages (Figure 9a), whereas the latter comprised of XCR1^+^ cDCs (Figure 9c). Consistent with previous data, labelled MRC1L‐B was rapidly internalized by macrophages [29]. Macrophages also internalized MHCII under these conditions, whereas MHCII staining on XCR1^+^ cDCs largely remained confined to the cell plasma membrane (Figure 9b).

### Salmonella Typhimurium interactions with cDCs

**FIGURE 10:**
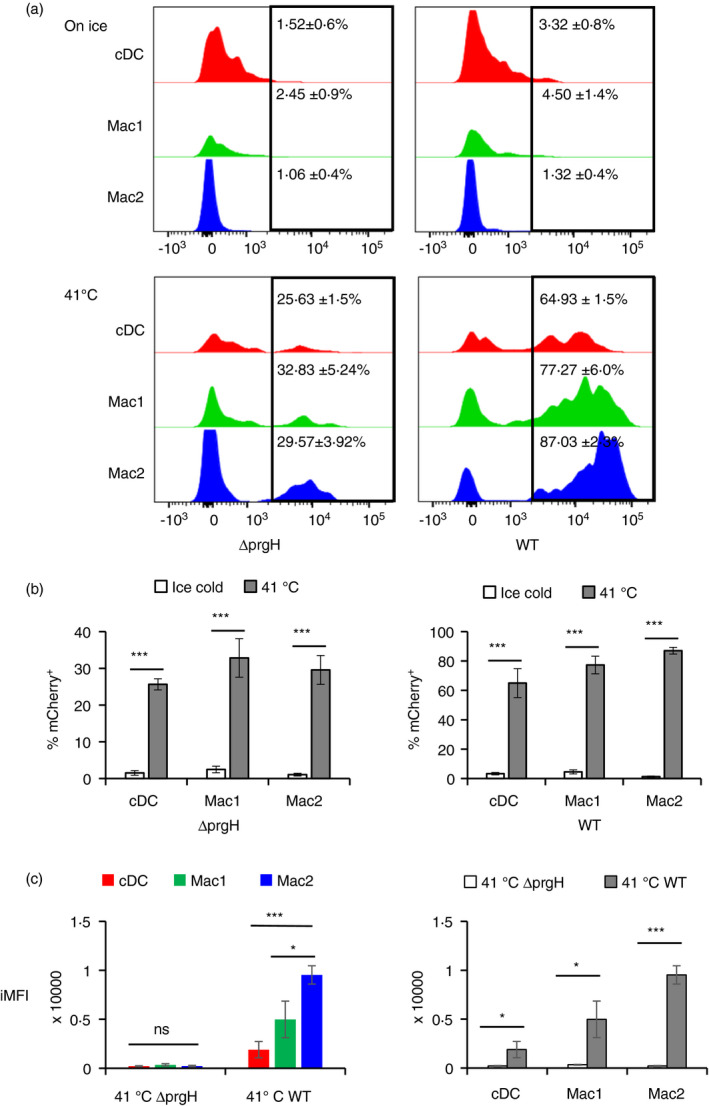
Flow cytometric analysis of single, live splenic cells from 11‐week‐old *CSF1R*‐eGFP^+^ birds (n = 3) incubated with *Salmonella* Typhimurium strain ST4/74 nal^R^ (wild‐type) or an isogenic mutant which lacks *prgH* (Δ*prgH*). Flow cytometric gating strategy to identify the three cell MPS populations was the same as in Figure 4. *CSF1R*‐eGFP^+^FLT3^HI^ = ‘cDC’, *CSF1R*‐eGFP^+^FLT3^LOW^ = ‘Mac1’) and *CSF1R*‐eGFP^+^FLT3^−^ = ‘Mac2’. (a and b) Frequency of cells expressing *Salmonella*‐mCherry in each population (the mean ±SD). The adhesion and entry of *Salmonella* into MPS cells were carried out at 41°C, with duplicated experiment carried out on ice as controls. Gentamicin also used to kill bacteria extracellular bacteria; hence, only intracellular bacteria were detected by their expression of mCherry by flow cytometry. (c) Comparisons of the integrated mean fluorescence intensity (iMFI) of *Salmonella*‐mCherry^+^ cells across different populations. Statistical analysis was conducted using two‐tailed unpaired t‐test with Welch correction. Statistical significance was always defined as follows: ∗, *p* < 0·05; ∗∗, *p* < 0·01; and ∗∗∗, *p* < 0·001

*Salmonella enterica* serovars that are frequently isolated from chickens, such as Typhimurium and Enteritidis, also infect humans and are a major public health concern. Much of our knowledge of *Salmonella* host–pathogen interactions derives from mouse models of typhoid fever and enterocolitis. Basic knowledge of the interaction between these zoonotic bacteria and immune cells is largely lacking in avian studies. After oral infection of chickens, *Salmonella* can be isolated from a number of peripheral organs, including the spleen, liver and reproductive tract and can be found residing within splenic macrophages in asymptomatic chickens. However, it is not known whether the presence of *Salmonella* with chicken splenic macrophages represents phagocytosis by this cell type or whether *Salmonella* exhibits a specific tropism for this cell type. To differentiate phagocytosis from active invasion of cells by *Salmonella* Typhimurium, *CSF1R*‐eGFP^+^ splenic MPS cells were exposed *in vitro* to either wild‐type (WT) or a non‐invasive Δ*prgH* mutant *S*. Typhimurium at a multiplicity of infection of five. Both bacterial strains expressed the fluorescent protein mCherry [32]. The cDC and macrophages were gated based on levels of FLT3 expression. Firstly, the percentage of mCherry^+^ cells were identified for each population (Figure 10a,b), and then, iMFI was calculated by multiplying the relative percentage of mCherry^+^ cells with the geometric mean fluorescence intensity (MFI) of mCherry^+^ populations. The negligible mCherry fluorescence detected in cells when the assay was carried out on ice indicates that lowering the temperature below 4°C inhibited the bacterial binding and internalization in all chicken cell types examined. At 41°C, each MPS population internalized the non‐invasive mutant or wild‐type strain (Figure 10). There was no significant difference in iMFI between the three MPS populations when treated with the Δ*prgH* mutant strain, suggesting that chicken cDCs can phagocytose *Salmonella* as efficiently macrophages. In contrast, based on iMFI as a proxy for the number of intracellular bacteria per cell, there was a preference for invasion by WT *Salmonella* of the *CSF1R*‐eGFP^+^ FLT3^−^macrophage subset compared to both the *CSF1R*‐eGFP^+^ FLT3^HI^ cDC or the *CSF1R*‐eGFP^+^ FLT3^LOW^ macrophage subset. As the iMFI was significantly higher in all MPS cell types when wild‐type bacteria were used (Figure 10c), this indicates that active invasion of chicken splenic MPS mediated by type III secretion system 1 is the major mechanism of *S*. Typhimurium intracellular uptake.

## DISCUSSION

The present study provides the first phenotypic and functional characterization of chicken cDCs by combining novel immunological tools to chicken cDC surface markers and the *CSF1R*‐transgenic reporter chicken. We identified the full‐length chicken *FLT3* transcript and expressed chicken FLT3 protein. We used a novel anti‐FLT3 Ab to identify and characterize FLT3^+^ cells in post‐hatch chickens. In the spleen FLT3^HI^, FLT3^LOW^ and FLT3^−^ subpopulations were identified, with the former population co‐expressing XCR1 and lacking expression of the commonly used chicken macrophage marker, MRC1L‐B (KUL01 antigen). We propose that this FLT3^HI^ XCR1^+^ cell subset likely represents the sole *bona fide* chicken cDC population. This work also highlights the value of using fluorescently labelled ligands to cell surface receptors, including the chemokine XCL1 and the growth factor CSF2, as an alternative to antibodies for analysing immune cell populations.

The MPS is comprised of monocytes, macrophages and dendritic cells. Traditionally, these cell subsets have been defined by morphology, phenotypical characteristics and function. More recently, it has been suggested that cells of the can classified primarily by their ontogeny and secondarily by their location, function and phenotype [13]. In this system, macrophages and monocyte‐derived cells are differentiated from dendritic cells, on the basis that the latter are derived from a common dendritic cell precursor [13, 15, 74]. Mammalian dendritic cells are further divided into plasmacytoid dendritic cells (pDC) and cDCs.

In early studies, antigen trapping chicken splenic dendritic cells were identified in the periphery of the splenic ellipsoid, surrounding the central arterioles of the white pulp and within germinal centres [75, 76]. Antigen complex trapping bursal secretory dendritic cells (BSDC), located within medulla of bursal B‐cell follicles, were also identified [77, 78, 79]. A polyclonal antibody to bovine S100 antigens and monoclonal antibody (CVI‐ChNL‐74·3) to an unknown antigen, also recognizes bursal BSDCs [80], as well as splenic ellipsoid associated cells (EAC) and follicular dendritic cells (FDCs) within germinal centres [81, 82]. Based on antigen trapping function and antibody staining, it has been suggested that EAC are the precursors to chicken FDCs in the spleen [83]. In mice, FDCs are derived from a non‐haematopoietic stromal cell precursor [83]. We have shown that chicken FDCs and BSDC express high levels of both the *CSF1R*‐transgene reporter and CSF1R protein [10]. This suggests that chicken FDCs and BSDC are part of the macrophage/monocyte lineage [10].

In the present study, FLT3^+^ MHCII^+^ cells were excluded from the splenic ellipsoids and germinal centres, as well as bursal B‐cell follicles. This indicates that chicken BSDCs and FDCs are not FLT3^+^ MHCII^+^ cDCs, Splenic FLT3^+^ MHCII^+^ cDCs were abundant and restricted to the red pulp and periarterial lymphoid sheath (PALS) areas of the spleen. Anti‐chFLT3 also specifically stained cells in the splenic ellipsoid capillary, suggesting a possible role in regulating endothelial cell development. In a previous study, we identified a cell population enriched for the expression of cDC associated genes, for example (*FLT3*, *BLB2* (encoding the chicken MHC class II beta chain 2), *XCR1*, *CADM1*, *CIITA*, *CD74* and *IRF8*) and hypothesized that in comparison with the mammalian liver, chicken cDCs were relatively abundant [27]. The present study agrees with this hypothesis, as we show that FLT3^+^ MHCII^+^ cells (identified as chicken cDCs) were abundant in the liver, mainly concentrated around liver blood vessels. In contrast MRC1L‐B^+^ macrophages are not clustered around liver blood vessels (this study, [27]). These data indicate that chicken FLT3^+^ cDCs are located in different anatomical locations to chicken FDCs, BSDCs and liver macrophage populations Despite anatomical differences between the structure of the murine and chicken spleen (mainly the lack of PWP in the former case), the location of FLT3^+^ MHCII^+^ cDCs in the chicken spleen appears to be similar to the reported location of XCR1^+^ cDCs in the murine spleen, which are located in T‐cell areas, such as the PALS, and red pulp of the spleen [84, 85].

Mammalian cDCs consist of two subsets: cDC1 and cDC2. Each cDC subset exhibits functional specialization, which is further influenced by tissue microenvironment predominately in non‐lymphoid organs [12, 14, 15, 16, 72]. The murine cDC1 subset is specialized for cross‐presentation of antigens and induction on Th1 responses, whereas the cDC2 subset is specialized for the induction of Th2 and Th17 immune responses [86]. Using transcriptomic approaches, a cDC subset, proposed to be equivalent to the mammalian XCR1^+^ cDC1 subset, was identified in the *CSF1R*‐transgene^+^ population in the chicken spleen, liver and lungs [26, 27, 28, 29, 87]. In the present study, all splenic FLT3^HI^ cells expressed XCR1. In support of previous work, the vast majority (~80%) of FLT3^HI^ XCR1^+^ cDCs were *CSF1R*‐eGFP^+^. No differences were observed in cell surface marker expression between the *CSF1R*‐eGFP^+^ and *CSF1R*‐eGFP^−^ FLT3^HI^ XCR1^+^ cDCs, and both subsets expressed the same level of CSF1R protein on the cell surface. This suggests that the level of *CSF1R*‐eGFP transgene expression is not related to cell maturation or activation status. In combination with FLT3 and/or XCR1 staining, *CSF1R*‐eGFP expression is very useful for characterizing the phenotype of chicken cDCs.

There is no evidence in the FLT3^HI^ cell population that chickens have the equivalent of the mammalian cDC2 subset [87, 88], which in any case is difficult to distinguish from a macrophage on the basis of markers or transcriptomic profile [47]. As low‐to‐intermediate expression of FLT3 was observed on both *CSF1R*‐eGFP negative and positive populations, we cannot rule out the possibility that other chicken DC populations (e.g. pDCs) exist in the FLT3 low/intermediate fraction. Further immunological tools will be required to determine if this is the case.

Despite the shared expression of XCR1^+^, there are several major differences between chicken cDCs and the mammalian cDC1 subset. Most obviously, they are abundant in chicken lymphoid tissues and both FLT3 and XCR1 mRNA are readily detected in total spleen and caecal tonsil mRNA [44]. Indeed, in the liver, the putative cDC identified with the Csf1r‐mApple transgene that expressed FLT3 and XCR1 mRNA were of similar abundance to Kupffer cells [27]. In chicken, the cDCs express high levels of CSF1R on their surface, but not CSF2R (Figure 7c). CSF1R is archetypal macrophage lineage specific receptor, being the receptor for the macrophage growth factors CSF1 and IL‐34. In the chicken, as in mammals, we have found that in addition to the macrophages and monocytes, CSF1R mRNA and reporter genes are expressed by granulocytes [10]. An additional novel function in birds is suggested by expression in epithelial‐derived antigen sampling M‐cells [32]. Nevertheless, the relative lack of CSF1R expression has been used as selection criteria for identifying the cDC1 subsets [47, 89]. We are currently generating both CSF1R‐ and FLT3‐deficient chickens to directly address the requirement for these growth factors in the development and function of chicken cDCs.

In this study, we identify the *CSF1R*‐eGFP^+^ XCR1^−^ FLT3^LOW^ subpopulation as a candidate for a unique chicken APC population. Expression of FLT3 suggests a DC origin, whereas expression of the classical monocyte/macrophage marker MRC1L‐B suggests that is subset is a specialized macrophage population. Chicken splenic MRC1L‐B^HI^ MHCII^LOW^ and MRC1L‐B^LOW^ MHCII^HI^ macrophage subsets have recently been characterized by Yu and colleagues [90]. Despite subset‐specific immune responses to lipopolysaccharide (LPS) stimulation, based on adoptive cell transfer experiments, both MRC1L‐B subsets were suggested to have a common monocytic origin. Taken together, these data suggest a macrophage/monocyte origin for the FLT3^LOW^ MRC1L‐B^LOW^ MHCII^HI^ *CSF1R*‐transgene^+^ subset identified in this study. Irrespective of these ontological origins, their high‐level expression of MHCII is consistent with the identification of this subset as chicken APCs.

*Salmonella enterica* causes disease in poultry and *serovars* that are frequently isolated from chickens, such as Typhimurium and Enteritidis, also infect humans and are a major public health concern. Despite this, very little is known regarding *Salmonella* chicken cell tropism. After infection with *Salmonella* Pullorum, intracellular bacteria were detected in splenic MRC1L‐B^+^ macrophages [91], and more recently, we demonstrated that *Salmonella Typhimurium* invading the bursa of Fabricius follicle‐associated epithelium are found within TIM4^+^ macrophages [32]. Furthermore, as pathogenic bacteria (e.g. *Mycobacterium tuberculosis*, *Salmonella* Typhimurium, *Yersinia enterocolitica* or *Escherichia coli*) evade immune response by targeting host cells for intracellular invasion, the presence of intracellular bacteria does not distinguish between phagocytosis of bacteria by the host cell and active invasion of host cells by intracellular pathogens. To distinguish between these outcomes, we compared the uptake of fluorescently labelled invasive wild‐type and non‐invasive ΔprgH Salmonella typhimurium by splenic *CSF1R*‐transgene expressing cells. FLT3^HI^, FLT3^LOW^ and FLT3^−^ *CSF1R*‐transgene expressing subpopulations expressing subpopulations were equally capable of phagocytosing ΔprgH *S*. Typhimurium. We found that *S*. Typhimurium also actively invaded all three subsets of splenic APC cells, but to a greater extent in the FLT3^LOW^ and FLT3^−^ *CSF1R*‐transgene expressing subpopulations. As both these subsets express MRC1L‐B, these data are consistent with the location of intracellular *Salmonella* observed in splenic MRC1L‐B^+^ macrophages [91] after *in vivo* challenge. Nevertheless, the chicken FLT3^HI^ XCR1^+^ cDCs were clearly also susceptible to pathogen invasion. In mammals, *Salmonella* infection modulates dendritic cell survival and function in a subset‐specific manner [92] and uptake of *Salmonella* interferes with antigen presentation [93, 94]. Our data paint a complex picture of *Salmonella* interactions with different APC subsets during infection in chickens, with *Salmonella* preferentially targeting macrophages, but also invading cDCs and potentially interfering with cDC function. To develop novel intervention strategies to control *Salmonella* and other bacterial pathogens in chickens, knowledge on the specific *in vivo* roles of chicken cDCs and other MPS subsets during infection and immunity will be required. We are currently generating chicken cDC knockout lines, which will enable these questions to be addressed.

Consistent with the lack of CSF2R expression on chicken cDCs, we show that the chicken CSF2‐differentiated bone marrow‐derived cultures do not contain cDCs. Despite the widespread use of BMDC as a proxy for *in vivo* DC populations, both CSF2‐differentiated chicken and murine ‘BMDCs’ are comprised of a heterogeneous population of cells [95, 96, 97] and there is an ongoing debate about how representative *in vitro* grown bone marrow‐derived DCs are of their *in vivo* counterparts [96]. Murine CSF2 bone marrow cultures have been shown to contain both FLT3^+^ cDCs and monocyte‐derived macrophages [96]. We show here that ChBMDCs also are a heterogeneous population that consist of two major MRC1L‐B^+^ subsets, neither of which expresses FLT3 or XCR1. Therefore, the use of chBMDCs as functional proxies for cDCs needs to be treated with caution.

## CONCLUSIONS

We present here a detailed analysis of chicken splenic APCs, using a range of novel immunological reagents including anti‐FLT3 monoclonal antibody; XCL1^AF647^ and CSF2^AF647^ which allowed efficient and precise identification of *bona fide* chicken cDCs. XCR1 expression was confined to the FLT3^HI^ expressing cells. These cells expressed high levels of MHCII and exhibited a typical dendritic cell morphology in tissue sections and *in vitro* culture. By immunofluorescence staining we could differentiate FLT3^+^ MHCII^+^ cells from previously identified chicken dendritic cell populations (e.g. FDCs and BSDC) and macrophages, suggesting different ontological origins between chicken cDCs and other cells of the chicken MPS. Consistent with previous studies which have identified chicken cDCs on the basis of transcriptional signature [26, 27, 29, 87, 88], these data indicate that the XCR1^+^ cDC lineage arose before the diversion of the avian and mammalian lineages (last common ancestor ~320 million years ago) and has retained many features in common in both lineages [26, 74, 75], suggesting a conserved role in uptake of dead cells and the cross‐presentation of antigens [26, 87, 88]. Unlike mouse DC, which lack Timd4 mRNA [47], chicken cDC also express TIM4, a receptor for apoptotic cells [27]. XCL1 ‘tags’ dead cells, suggesting a role for XCR1‐XCL1 interactions in the direct recognition of dead cells by XCR1^+^ cDCs, as well as the role in cell chemotaxis. We found significance divergence in the expression of growth factor receptors between the chicken and mammalian XCR1^+^ cDCs. Chicken cDCs express high levels of CSF1R and low levels of CSF2R, while the converse is true for the mammalian XCR1^+^ cDC1 subset. As such, CSF2‐differentiated chicken bone marrow‐derived cultures do not contain cDCs. We show that despite the preferential invasion of macrophages by *Salmonella* Typhimurium, chicken cDCs are equality effective at the uptake of live non‐invasive *Salmonella* Typhimurium. In mammals, XCR1^+^ cDCs are believed to be specialized for the cross‐presentation of antigens and the generation of cytotoxic T‐cell responses [18, 21, 98]. We now have the tools to investigate the relative contribution of chicken XCR1^+^ cDCs to host defence and the development of acquired immunity.

## CONFLICT OF INTEREST

The authors declare no competing interests in reference to this manuscript.

## AUTHOR CONTRIBUTIONS

ZW, TH, CCU, JM and AB performed the experiments. ZW, TH, CCU, JM, MPS and AB analysed the data. PK and AB designed the study. ZW, HS, DAH, MPS and AB wrote and edited the manuscript.

## PERMISSION TO REPRODUCE

None.

## Supporting information

Fig S1

Fig S2

Fig S3

Fig S4

## References

[1] Liu YJ . Dendritic cell subsets and lineages, and their functions in innate and adaptive immunity. Cell. 2001;106:259–62. 11509173 10.1016/s0092-8674(01)00456-1

[2] Merad M , Sathe P , Helft J , Miller J , Mortha A . The dendritic cell lineage: ontogeny and function of dendritic cells and their subsets in the steady state and the inflamed setting. Annu Rev Immunol. 2013;31:563–604. 23516985 10.1146/annurev-immunol-020711-074950 PMC3853342

[3] Mildner A , Jung S . Development and function of dendritic cell subsets. Immunity. 2014;40:642–56. 24837101 10.1016/j.immuni.2014.04.016

[4] Anderson DA 3rd , Dutertre CA , Ginhoux F , Murphy KM . Genetic models of human and mouse dendritic cell development and function. Nat Rev Immunol. 2021;21:101–15. 32908299 10.1038/s41577-020-00413-x PMC10955724

[5] Sichien D , Lambrecht BN , Guilliams M , Scott CL . Development of conventional dendritic cells: from common bone marrow progenitors to multiple subsets in peripheral tissues. Mucosal Immunol. 2017;10:831–44. 28198365 10.1038/mi.2017.8

[6] Cabeza‐Cabrerizo M , van Blijswijk J , Wienert S , Heim D , Jenkins RP , Chakravarty P , et al. Tissue clonality of dendritic cell subsets and emergency DCpoiesis revealed by multicolor fate mapping of DC progenitors. Sci Immunol. 2019;4:eaaw1941. 30824528 10.1126/sciimmunol.aaw1941 PMC6420147

[7] Alvarez D , Vollmann EH , von Andrian UH . Mechanisms and consequences of dendritic cell migration. Immunity. 2008;29:325–42. 18799141 10.1016/j.immuni.2008.08.006 PMC2818978

[8] Casteleyn C , Doom M , Lambrechts E , Van den Broeck W , Simoens P , Cornillie P . Locations of gut‐associated lymphoid tissue in the 3‐month‐old chicken: a review. Avian Pathol. 2010;39:143–50. 20544418 10.1080/03079451003786105

[9] Befus AD , Johnston N , Leslie GA , Bienenstock J . Gut‐associated lymphoid tissue in the chicken. I. Morphology, ontogeny, and some functional characteristics of Peyer's patches. J Immunol. 1980;125:2626–32. 7430642

[10] Balic A , Garcia‐Morales C , Vervelde L , Gilhooley H , Sherman A , Garceau V , et al. Visualisation of chicken macrophages using transgenic reporter genes: insights into the development of the avian macrophage lineage. Development. 2014;141:3255–65. 25063453 10.1242/dev.105593 PMC4197536

[11] Kaiser P . The long view: a bright past, a brighter future? Forty years of chicken immunology pre‐ and post‐genome. Avian Pathol. 2012;41:511–8. 23237363 10.1080/03079457.2012.735359

[12] Karsunky H , Merad M , Cozzio A , Weissman IL , Manz MG . Flt3 ligand regulates dendritic cell development from Flt3+ lymphoid and myeloid‐committed progenitors to Flt3+ dendritic cells in vivo. J Exp Med. 2003;198:305–13. 12874263 10.1084/jem.20030323 PMC2194067

[13] Guilliams M , Ginhoux F , Jakubzick C , Naik SH , Onai N , Schraml BU , et al. Dendritic cells, monocytes and macrophages: a unified nomenclature based on ontogeny. Nat Rev Immunol. 2014;14:571–8. 25033907 10.1038/nri3712 PMC4638219

[14] D'Amico A , Wu L . The early progenitors of mouse dendritic cells and plasmacytoid predendritic cells are within the bone marrow hemopoietic precursors expressing Flt3. J Exp Med. 2003;198:293–303. 12874262 10.1084/jem.20030107 PMC2194069

[15] Onai N , Obata‐Onai A , Schmid MA , Ohteki T , Jarrossay D , Manz MG . Identification of clonogenic common Flt3+M‐CSFR+ plasmacytoid and conventional dendritic cell progenitors in mouse bone marrow. Nat Immunol. 2007;8:1207–16. 17922016 10.1038/ni1518

[16] Waskow C , Liu K , Darrasse‐Jeze G , Guermonprez P , Ginhoux F , Merad M , et al. The receptor tyrosine kinase Flt3 is required for dendritic cell development in peripheral lymphoid tissues. Nat Immunol. 2008;9:676–83. 18469816 10.1038/ni.1615 PMC2746085

[17] Liu K , Victora GD , Schwickert TA , Guermonprez P , Meredith MM , Yao K , et al. In vivo analysis of dendritic cell development and homeostasis. Science. 2009;324:392–7. 19286519 10.1126/science.1170540 PMC2803315

[18] den Haan JM , Lehar SM , Bevan MJ . CD8(+) but not CD8(‐) dendritic cells cross‐prime cytotoxic T cells in vivo. J Exp Med. 2000;192:1685–96. 11120766 10.1084/jem.192.12.1685 PMC2213493

[19] Iyoda T , Shimoyama S , Liu K , Omatsu Y , Akiyama Y , Maeda Y , et al. The CD8+ dendritic cell subset selectively endocytoses dying cells in culture and in vivo. J Exp Med. 2002;195:1289–302. 12021309 10.1084/jem.20020161 PMC2193756

[20] Bedoui S , Whitney PG , Waithman J , Eidsmo L , Wakim L , Caminschi I , et al. Cross‐presentation of viral and self antigens by skin‐derived CD103+ dendritic cells. Nat Immunol. 2009;10:488–95. 19349986 10.1038/ni.1724

[21] Kroczek RA , Henn V . The role of XCR1 and its Ligand XCL1 in antigen cross‐presentation by murine and human dendritic cells. Front Immunol. 2012;3:14. 22566900 10.3389/fimmu.2012.00014 PMC3342032

[22] Kashem SW , Igyarto BZ , Gerami‐Nejad M , Kumamoto Y , Mohammed JA , Jarrett E , et al. Candida albicans morphology and dendritic cell subsets determine T helper cell differentiation. Immunity. 2015;42:356–66. 25680275 10.1016/j.immuni.2015.01.008 PMC4343045

[23] Linehan JL , Dileepan T , Kashem SW , Kaplan DH , Cleary P , Jenkins MK . Generation of Th17 cells in response to intranasal infection requires TGF‐beta1 from dendritic cells and IL‐6 from CD301b+ dendritic cells. Proc Natl Acad Sci U S A. 2015;112:12782–7. 26417101 10.1073/pnas.1513532112 PMC4611596

[24] Kumamoto Y , Hirai T , Wong PW , Kaplan DH , Iwasaki A . CD301b(+) dendritic cells suppress T follicular helper cells and antibody responses to protein antigens. Elife. 2016;5:e17979. 27657168 10.7554/eLife.17979 PMC5033605

[25] Ginhoux F , Guilliams M , Naik SH . Editorial: Dendritic cell and macrophage nomenclature and classification. Front Immunol. 2016;7:168. 27199991 10.3389/fimmu.2016.00168 PMC4852170

[26] Vu Manh TP , Marty H , Sibille P , Le Vern Y , Kaspers B , Dalod M , et al. Existence of conventional dendritic cells in *Gallus gallus* revealed by comparative gene expression profiling. J Immunol. 2014;192:4510–7. 24740508 10.4049/jimmunol.1303405

[27] Hu T , Wu Z , Bush SJ , Freem L , Vervelde L , Summers KM , et al. Characterization of subpopulations of chicken mononuclear phagocytes that express TIM4 and CSF1R. J Immunol. 2019;202:1186–99. 30626692 10.4049/jimmunol.1800504 PMC6436730

[28] Alber A , Morris KM , Bryson KJ , Sutton KM , Monson MS , Chintoan‐Uta C , et al. Avian pathogenic Escherichia coli (APEC) strain‐dependent immunomodulation of respiratory granulocytes and mononuclear phagocytes in CSF1R‐reporter transgenic chickens. Front Immunol. 2019;10:3055. 31998322 10.3389/fimmu.2019.03055 PMC6967599

[29] Sutton KM , Morris KM , Borowska D , Sang H , Kaiser P , Balic A , et al. Characterization of conventional dendritic cells and macrophages in the spleen using the CSF1R‐reporter transgenic chickens. Front Immunol. 2021;12:636436.

[30] Wu Z , Harne R , Chintoan‐Uta C , Hu TJ , Wallace R , MacCallum A , et al. Regulation and function of macrophage colony‐stimulating factor (CSF1) in the chicken immune system. Dev Comp Immunol. 2020;105:103586. 31870792 10.1016/j.dci.2019.103586 PMC6996135

[31] Garcia‐Morales C , Rothwell L , Moffat L , Garceau V , Balic A , Sang HM , et al. Production and characterisation of a monoclonal antibody that recognises the chicken CSF1 receptor and confirms that expression is restricted to macrophage‐lineage cells. Dev Comp Immunol. 2014;42:278–85. 24084378 10.1016/j.dci.2013.09.011

[32] Balic A , Chintoan‐Uta C , Vohra P , Sutton KM , Cassady‐Cain RL , Hu T , et al. Antigen sampling CSF1R‐expressing epithelial cells are the functional equivalents of mammalian M cells in the avian follicle‐associated epithelium. Front Immunol. 2019;10:2495. 31695701 10.3389/fimmu.2019.02495 PMC6817575

[33] Garceau V , Balic A , Garcia‐Morales C , Sauter KA , McGrew MJ , Smith J , et al. The development and maintenance of the mononuclear phagocyte system of the chick is controlled by signals from the macrophage colony‐stimulating factor receptor. BMC Biol. 2015;13:12. 25857347 10.1186/s12915-015-0121-9 PMC4369834

[34] Sutton K , Costa T , Alber A , Bryson K , Borowska D , Balic A , et al. Visualisation and characterisation of mononuclear phagocytes in the chicken respiratory tract using CSF1R‐transgenic chickens. Vet Res. 2018;49:104. 30305141 10.1186/s13567-018-0598-7 PMC6389226

[35] Smith SE , Gibson MS , Wash RS , Ferrara F , Wright E , Temperton N , et al. Chicken interferon‐inducible transmembrane protein 3 restricts influenza viruses and lyssaviruses in vitro. J Virol. 2013;87:12957–66. 24067955 10.1128/JVI.01443-13 PMC3838109

[36] Wu Z , Hu T , Rothwell L , Vervelde L , Kaiser P , Boulton K , et al. Analysis of the function of IL‐10 in chickens using specific neutralising antibodies and a sensitive capture ELISA. Dev Comp Immunol. 2016;63:206–12. 27108075 10.1016/j.dci.2016.04.016 PMC4947970

[37] Longo PA , Kavran JM , Kim MS , Leahy DJ . Transient mammalian cell transfection with polyethylenimine (PEI). Methods Enzymol. 2013;529:227–40. 24011049 10.1016/B978-0-12-418687-3.00018-5 PMC4012321

[38] Rothwell L , Hamblin A , Kaiser P . Production and characterisation of monoclonal antibodies specific for chicken interleukin‐2. Vet Immunol Immunopathol. 2001;83:149–60. 11730926 10.1016/s0165-2427(01)00391-9

[39] Avery S , Rothwell L , Degen WD , Schijns VE , Young J , Kaufman J , et al. Characterization of the first nonmammalian T2 cytokine gene cluster: the cluster contains functional single‐copy genes for IL‐3, IL‐4, IL‐13, and GM‐CSF, a gene for IL‐5 that appears to be a pseudogene, and a gene encoding another cytokine‐like transcript, KK34. J Interferon Cytokine Res. 2004;24:600–10. 15626157 10.1089/jir.2004.24.600

[40] Wu Z , Rothwell L , Young JR , Kaufman J , Butter C , Kaiser P . Generation and characterization of chicken bone marrow‐derived dendritic cells. Immunology. 2010;129:133–45. 19909375 10.1111/j.1365-2567.2009.03129.x PMC2807494

[41] Valdivia RH , Falkow S . Bacterial genetics by flow cytometry: rapid isolation of Salmonella typhimurium acid‐inducible promoters by differential fluorescence induction. Mol Microbiol. 1996;22:367–78. 8930920 10.1046/j.1365-2958.1996.00120.x

[42] Darrah PA , Patel DT , De Luca PM , Lindsay RW , Davey DF , Flynn BJ , et al. Multifunctional TH1 cells define a correlate of vaccine‐mediated protection against Leishmania major. Nat Med. 2007;13:843–50. 17558415 10.1038/nm1592

[43] Shooshtari P , Fortuno ES 3rd , Blimkie D , Yu M , Gupta A , Kollmann TR , et al. Correlation analysis of intracellular and secreted cytokines via the generalized integrated mean fluorescence intensity. Cytometry A. 2010;77:873–80. 20629196 10.1002/cyto.a.20943 PMC2930075

[44] Bush SJ , Freem L , MacCallum AJ , O'Dell J , Wu C , Afrasiabi C , et al. Combination of novel and public RNA‐seq datasets to generate an mRNA expression atlas for the domestic chicken. BMC Genom. 2018;19:594. 10.1186/s12864-018-4972-7 PMC6081845 30086717

[45] Matthews W , Jordan CT , Gavin M , Jenkins NA , Copeland NG , Lemischka IR . A receptor tyrosine kinase cDNA isolated from a population of enriched primitive hematopoietic cells and exhibiting close genetic linkage to c‐kit. Proc Natl Acad Sci U S A. 1991;88:9026–30. 1717995 10.1073/pnas.88.20.9026 PMC52644

[46] Miller G , Pillarisetty VG , Shah AB , Lahrs S , DeMatteo RP . Murine Flt3 ligand expands distinct dendritic cells with both tolerogenic and immunogenic properties. J Immunol. 2003;170:3554–64. 12646617 10.4049/jimmunol.170.7.3554

[47] Summers KM , Bush SJ , Hume DA . Network analysis of transcriptomic diversity amongst resident tissue macrophages and dendritic cells in the mouse mononuclear phagocyte system. PLoS Biol. 2020;18:e3000859. 33031383 10.1371/journal.pbio.3000859 PMC7575120

[48] Houssaint E , Diez E , Pink JR . Ontogeny and tissue distribution of the chicken Bu‐1a antigen. Immunology. 1987;62:463–70. 3499381 PMC1454130

[49] Mast J , Goddeeris BM , Peeters K , Vandesande F , Berghman LR . Characterisation of chicken monocytes, macrophages and interdigitating cells by the monoclonal antibody KUL01. Vet Immunol Immunopathol. 1998;61:343–57. 9613446 10.1016/s0165-2427(97)00152-9

[50] Bachem A , Hartung E , Guttler S , Mora A , Zhou X , Hegemann A , et al. Expression of XCR1 Characterizes the Batf3‐Dependent Lineage of Dendritic Cells Capable of Antigen Cross‐Presentation. Front Immunol. 2012;3:214. 22826713 10.3389/fimmu.2012.00214 PMC3399095

[51] Crozat K , Guiton R , Contreras V , Feuillet V , Dutertre CA , Ventre E , et al. The XC chemokine receptor 1 is a conserved selective marker of mammalian cells homologous to mouse CD8alpha+ dendritic cells. J Exp Med. 2010;207:1283–92. 20479118 10.1084/jem.20100223 PMC2882835

[52] Crozat K , Tamoutounour S , Vu Manh TP , Fossum E , Luche H , Ardouin L , et al. Cutting edge: expression of XCR1 defines mouse lymphoid‐tissue resident and migratory dendritic cells of the CD8alpha+ type. J Immunol. 2011;187:4411–5. 21948982 10.4049/jimmunol.1101717

[53] Fossum E , Grodeland G , Terhorst D , Tveita AA , Vikse E , Mjaaland S , et al. Vaccine molecules targeting Xcr1 on cross‐presenting DCs induce protective CD8+ T‐cell responses against influenza virus. Eur J Immunol. 2015;45:624–35. 25410055 10.1002/eji.201445080

[54] Anselmo A , Mazzon C , Borroni EM , Bonecchi R , Graham GJ , Locati M . Flow cytometry applications for the analysis of chemokine receptor expression and function. Cytometry A. 2014;85:292–301. 24464630 10.1002/cyto.a.22439

[55] Le Brocq ML , Fraser AR , Cotton G , Woznica K , McCulloch CV , Hewitt KD , et al. Chemokines as novel and versatile reagents for flow cytometry and cell sorting. J Immunol. 2014;192:6120–30. 24850722 10.4049/jimmunol.1303371 PMC4821367

[56] Schulz O , Reis e Sousa C . Cross‐presentation of cell‐associated antigens by CD8alpha+ dendritic cells is attributable to their ability to internalize dead cells. Immunology. 2002;107:183–9. 12383197 10.1046/j.1365-2567.2002.01513.x PMC1782783

[57] Hartung E , Becker M , Bachem A , Reeg N , Jakel A , Hutloff A , et al. Induction of potent CD8 T cell cytotoxicity by specific targeting of antigen to cross‐presenting dendritic cells in vivo via murine or human XCR1. J Immunol. 2015;194:1069–79. 25520399 10.4049/jimmunol.1401903

[58] Jongbloed SL , Kassianos AJ , McDonald KJ , Clark GJ , Ju X , Angel CE , et al. Human CD141+ (BDCA‐3)+ dendritic cells (DCs) represent a unique myeloid DC subset that cross‐presents necrotic cell antigens. J Exp Med. 2010;207:1247–60. 20479116 10.1084/jem.20092140 PMC2882828

[59] Kroczek AL , Hartung E , Gurka S , Becker M , Reeg N , Mages HW , et al. Structure‐Function Relationship of XCL1 Used for in vivo Targeting of Antigen Into XCR1(+) Dendritic Cells. Front Immunol. 2018;9:2806. 30619244 10.3389/fimmu.2018.02806 PMC6300513

[60] Maaten L , Hinton G . Visualizing data using t‐SNE. J Machine Learning Res. 2008;9:2579–605.

[61] Auray G , Keller I , Python S , Gerber M , Bruggmann R , Ruggli N , et al. Characterization and transcriptomic analysis of porcine blood conventional and plasmacytoid dendritic cells reveals striking species‐specific differences. J Immunol. 2016;197:4791–806. 27837108 10.4049/jimmunol.1600672

[62] Ginhoux F , Liu K , Helft J , Bogunovic M , Greter M , Hashimoto D , et al. The origin and development of nonlymphoid tissue CD103+ DCs. J Exp Med. 2009;206:3115–30. 20008528 10.1084/jem.20091756 PMC2806447

[63] Hawley CA , Rojo R , Raper A , Sauter KA , Lisowski ZM , Grabert K , et al. Csf1r‐mApple transgene expression and ligand binding in vivo reveal dynamics of CSF1R expression within the mononuclear phagocyte system. J Immunol. 2018;200:2209–23. 29440354 10.4049/jimmunol.1701488 PMC5834790

[64] Metcalf D . Hematopoietic cytokines. Blood. 2008;111:485–91. 18182579 10.1182/blood-2007-03-079681 PMC2200848

[65] Balan S , Arnold‐Schrauf C , Abbas A , Couespel N , Savoret J , Imperatore F , et al. Large‐scale human dendritic cell differentiation revealing notch‐dependent lineage bifurcation and heterogeneity. Cell Rep. 2018;24:1902–15. 30110645 10.1016/j.celrep.2018.07.033 PMC6113934

[66] Greter M , Helft J , Chow A , Hashimoto D , Mortha A , Agudo‐Cantero J , et al. GM‐CSF controls nonlymphoid tissue dendritic cell homeostasis but is dispensable for the differentiation of inflammatory dendritic cells. Immunity. 2012;36:1031–46. 22749353 10.1016/j.immuni.2012.03.027 PMC3498051

[67] Hamilton JA . Colony‐stimulating factors in inflammation and autoimmunity. Nat Rev Immunol. 2008;8:533–44. 18551128 10.1038/nri2356

[68] Martinez‐Moczygemba M , Huston DP . Biology of common beta receptor‐signaling cytokines: IL‐3, IL‐5, and GM‐CSF. J Allergy Clin Immunol. 2003;112:653–65. 14564341 10.1016/S0091

[69] Rosas M , Gordon S , Taylor PR . Characterisation of the expression and function of the GM‐CSF receptor alpha‐chain in mice. Eur J Immunol. 2007;37:2518–28. 17694571 10.1002/eji.200636892 PMC2699419

[70] Bowers WE , Berkowitz MR . Differentiation of dendritic cells in cultures of rat bone marrow cells. J Exp Med. 1986;163:872–83. 3512761 10.1084/jem.163.4.872 PMC2188067

[71] Son YI , Egawa S , Tatsumi T , Redlinger RE Jr , Kalinski P , Kanto T . A novel bulk‐culture method for generating mature dendritic cells from mouse bone marrow cells. J Immunol Methods. 2002;262:145–57. 11983228 10.1016/s0022-1759(02)00013-3

[72] Freem L , Summers KM , Gheyas AA , Psifidi A , Boulton K , MacCallum A , et al. Analysis of the progeny of sibling matings reveals regulatory variation impacting the transcriptome of immune cells in commercial chickens. Front Genet. 2019;10:1032. 31803225 10.3389/fgene.2019.01032 PMC6870463

[73] Steinman RM , Cohn ZA . Identification of a novel cell type in peripheral lymphoid organs of mice. I. Morphology, quantification, tissue distribution. J Exp Med. 1973;137:1142–62. 4573839 10.1084/jem.137.5.1142 PMC2139237

[74] Naik SH , Sathe P , Park HY , Metcalf D , Proietto AI , Dakic A , et al. Development of plasmacytoid and conventional dendritic cell subtypes from single precursor cells derived in vitro and in vivo. Nat Immunol. 2007;8:1217–26. 17922015 10.1038/ni1522

[75] White RG , Henderson DC , Eslami MB , Neilsen KH . Localization of a protein antigen in the chicken spleen. Effect of various manipulative procedures on the morphogenesis of the germinal centre. Immunology. 1975;28:1–21. 46839 PMC1445742

[76] Eikelenboom P , Kroese FG , van Rooijen N . Immune complex‐trapping cells in the spleen of the chicken. Enzyme histochemical and ultrastructural aspects. Cell Tissue Res. 1983;231:377–86. 6342811 10.1007/BF00222188

[77] Olah I , Glick B . Secretory cell in the medulla of the bursa of Fabricius. Experientia. 1978;34:1642–3. 729745 10.1007/BF02034727

[78] Glick B . The ontogeny and microenvironment of the avian thymus and bursa of Fabricius: contribution of specialized cells to the avian immune response. Adv Vet Sci Comp Med. 1985;30:67–90. 3911760

[79] Glick B , Olah I . Bursal secretory dendritic‐like cell: a microenvironment issue. Poult Sci. 1993;72:1262–6. 8346151 10.3382/ps.0721262

[80] Nagy N , Bodi I , Olah I . Avian dendritic cells: Phenotype and ontogeny in lymphoid organs. Dev Comp Immunol. 2016;58:47–59. 26751596 10.1016/j.dci.2015.12.020

[81] Jeurissen SH , Claassen E , Janse EM . Histological and functional differentiation of non‐lymphoid cells in the chicken spleen. Immunology. 1992;77:75–80. 1398767 PMC1421597

[82] Jeurissen SH . The role of various compartments in the chicken spleen during an antigen‐specific humoral response. Immunology. 1993;80:29–33. 8244460 PMC1422104

[83] Igyarto BZ , Magyar A , Olah I . Origin of follicular dendritic cell in the chicken spleen. Cell Tissue Res. 2007;327:83–92. 16941124 10.1007/s00441-006-0250-0

[84] Yamazaki C , Sugiyama M , Ohta T , Hemmi H , Hamada E , Sasaki I , et al. Critical roles of a dendritic cell subset expressing a chemokine receptor, XCR1. J Immunol. 2013;190:6071–82. 23670193 10.4049/jimmunol.1202798

[85] Shimizu K , Asakura M , Shinga J , Sato Y , Kitahara S , Hoshino K , et al. Invariant NKT cells induce plasmacytoid dendritic cell (DC) cross‐talk with conventional DCs for efficient memory CD8+ T cell induction. J Immunol. 2013;190:5609–19. 23630347 10.4049/jimmunol.1300033

[86] Durai V , Murphy KM . Functions of murine dendritic cells. Immunity. 2016;45:719–36. 27760337 10.1016/j.immuni.2016.10.010 PMC5145312

[87] Vu Manh TP , Elhmouzi‐Younes J , Urien C , Ruscanu S , Jouneau L , Bourge M , et al. Defining mononuclear phagocyte subset homology across several distant warm‐blooded vertebrates through comparative transcriptomics. Front Immunol. 2015;6:299. 26150816 10.3389/fimmu.2015.00299 PMC4473062

[88] Vu Manh TP , Bertho N , Hosmalin A , Schwartz‐Cornil I , Dalod M . Investigating evolutionary conservation of dendritic cell subset identity and functions. Front Immunol. 2015;6:260. 26082777 10.3389/fimmu.2015.00260 PMC4451681

[89] MacDonald KP , Rowe V , Bofinger HM , Thomas R , Sasmono T , Hume DA , et al. The colony‐stimulating factor 1 receptor is expressed on dendritic cells during differentiation and regulates their expansion. J Immunol. 2005;175:1399–405. 16034075 10.4049/jimmunol.175.3.1399

[90] Yu K , Gu MJ , Pyung YJ , Song KD , Park TS , Han SH , et al. Characterization of splenic MRC1(hi)MHCII(lo) and MRC1(lo)MHCII(hi) cells from the monocyte/macrophage lineage of White Leghorn chickens. Vet Res. 2020;51:73. 32460863 10.1186/s13567-020-00795-9 PMC7251834

[91] Wigley P , Berchieri A Jr , Page KL , Smith AL , Barrow PA . Salmonella enterica serovar Pullorum persists in splenic macrophages and in the reproductive tract during persistent, disease‐free carriage in chickens. Infect Immun. 2001;69:7873–9. 11705970 10.1128/IAI.69.12.7873-7879.2001 PMC98884

[92] Kirby AC , Yrlid U , Svensson M , Wick MJ . Differential involvement of dendritic cell subsets during acute Salmonella infection. J Immunol. 2001;166:6802–11. 11359839 10.4049/jimmunol.166.11.6802

[93] Cheminay C , Mohlenbrink A , Hensel M . Intracellular Salmonella inhibit antigen presentation by dendritic cells. J Immunol. 2005;174:2892–9. 15728500 10.4049/jimmunol.174.5.2892

[94] Tobar JA , Gonzalez PA , Kalergis AM . Salmonella escape from antigen presentation can be overcome by targeting bacteria to Fc gamma receptors on dendritic cells. J Immunol. 2004;173:4058–65. 15356155 10.4049/jimmunol.173.6.4058

[95] Bottcher JP , Zelenay S , Rogers NC , Helft J , Schraml BU , Reis e Sousa C Oncogenic transformation of dendritic cells and their precursors leads to rapid cancer development in mice. J Immunol. 2015;195:5066–76. 26459350 10.4049/jimmunol.1500889 PMC4635568

[96] Helft J , Bottcher J , Chakravarty P , Zelenay S , Huotari J , Schraml BU , et al. GM‐CSF mouse bone marrow cultures comprise a heterogeneous population of CD11c(+)MHCII(+) macrophages and dendritic cells. Immunity. 2015;42:1197–211. 26084029 10.1016/j.immuni.2015.05.018

[97] van den Biggelaar R , Arkesteijn GJA , Rutten V , van Eden W , Jansen CA . In vitro chicken bone marrow‐derived dendritic cells comprise subsets at different states of maturation. Front Immunol. 2020;11:141. 32174908 10.3389/fimmu.2020.00141 PMC7054383

[98] Dorner BG , Dorner MB , Zhou X , Opitz C , Mora A , Guttler S , et al. Selective expression of the chemokine receptor XCR1 on cross‐presenting dendritic cells determines co‐operation with CD8+ T cells. Immunity. 2009;31:823–33. 19913446 10.1016/j.immuni.2009.08.027

