# Supplementary material for: Development of novel reagents to chicken FLT3, XCR1 and CSF2R for the identification and characterization of avian conventional dendritic cells
Source: Immunology. 2021 Nov 30;165(2):171–94. doi: 10.1111/imm.13426 (PMC10357484; doi:10.1111/imm.13426)
Supplement: Supplementary file 1 — Fig S1 [file IMM-165-171-s004.pptx]

## Slide 1
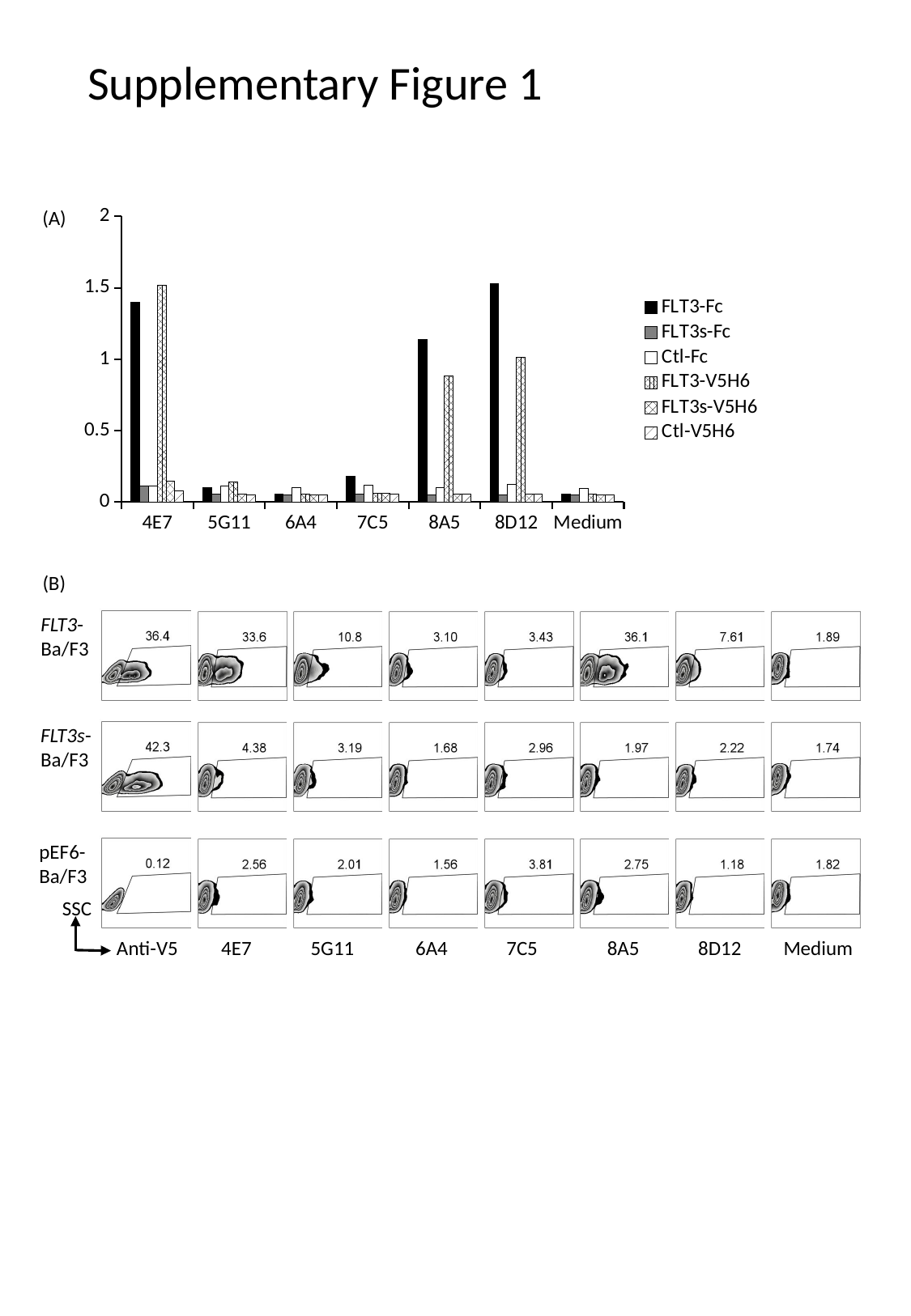

Supplementary Figure 1
(A)
### Chart
| Category | FLT3-Fc | FLT3s-Fc | Ctl-Fc | FLT3-V5H6 | FLT3s-V5H6 | Ctl-V5H6 |
|---|---|---|---|---|---|---|
| 4E7 | 1.399 | 0.111 | 0.111 | 1.52 | 0.146 | 0.076 |
| 5G11 | 0.098 | 0.053 | 0.112 | 0.138 | 0.055 | 0.051 |
| 6A4 | 0.053 | 0.048 | 0.101 | 0.054 | 0.052 | 0.052 |
| 7C5 | 0.179 | 0.055 | 0.116 | 0.062 | 0.058 | 0.057 |
| 8A5 | 1.137 | 0.052 | 0.098 | 0.88 | 0.057 | 0.053 |
| 8D12 | 1.526 | 0.052 | 0.12 | 1.012 | 0.053 | 0.054 |
| Medium | 0.056 | 0.049 | 0.097 | 0.053 | 0.05 | 0.049 |(B)
FLT3-Ba/F3
FLT3s-Ba/F3
pEF6-Ba/F3
SSC
Anti-V5
4E7
5G11
6A4
7C5
8A5
8D12
Medium
