# Supplementary material for: Development of novel reagents to chicken FLT3, XCR1 and CSF2R for the identification and characterization of avian conventional dendritic cells
Source: Immunology. 2021 Nov 30;165(2):171–94. doi: 10.1111/imm.13426 (PMC10357484; doi:10.1111/imm.13426)
Supplement: Supplementary file 2 — Fig S2 [file IMM-165-171-s003.pptx]

## Slide 1
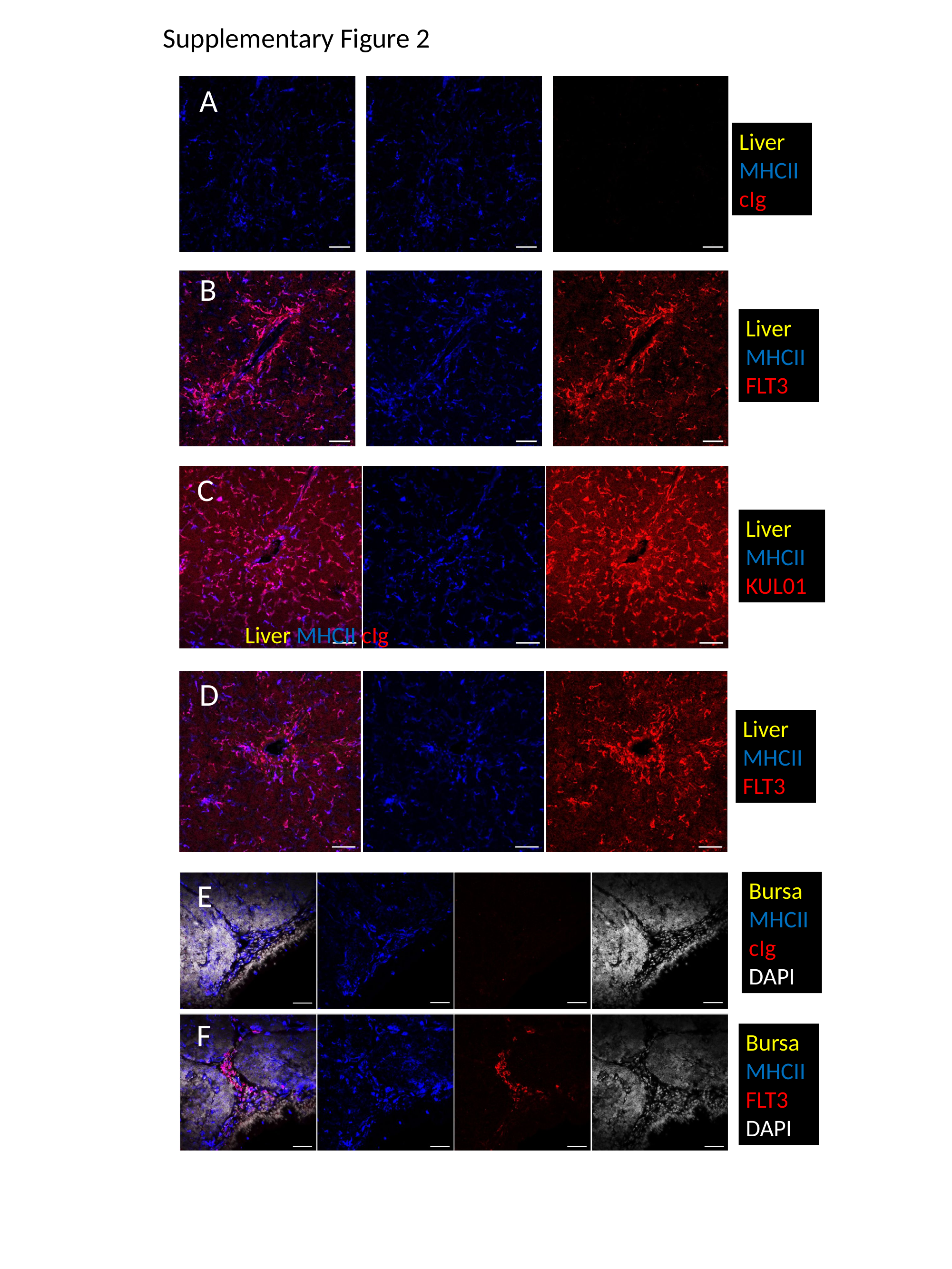

Supplementary Figure 2
A
Liver MHCII cIg
B
Liver MHCII FLT3
C
Liver MHCII KUL01
Liver MHCII cIg
D
Liver MHCII FLT3
E
BursaMHCII
cIg
DAPI
F
Bursa MHCII
FLT3
DAPI
