# Supplementary figures and images for: Development of novel reagents to chicken FLT3, XCR1 and CSF2R for the identification and characterization of avian conventional dendritic cells
Source: Immunology. 2021 Nov 30;165(2):171–94. doi: 10.1111/imm.13426 (PMC10357484; doi:10.1111/imm.13426)

Supplementary Figure 3

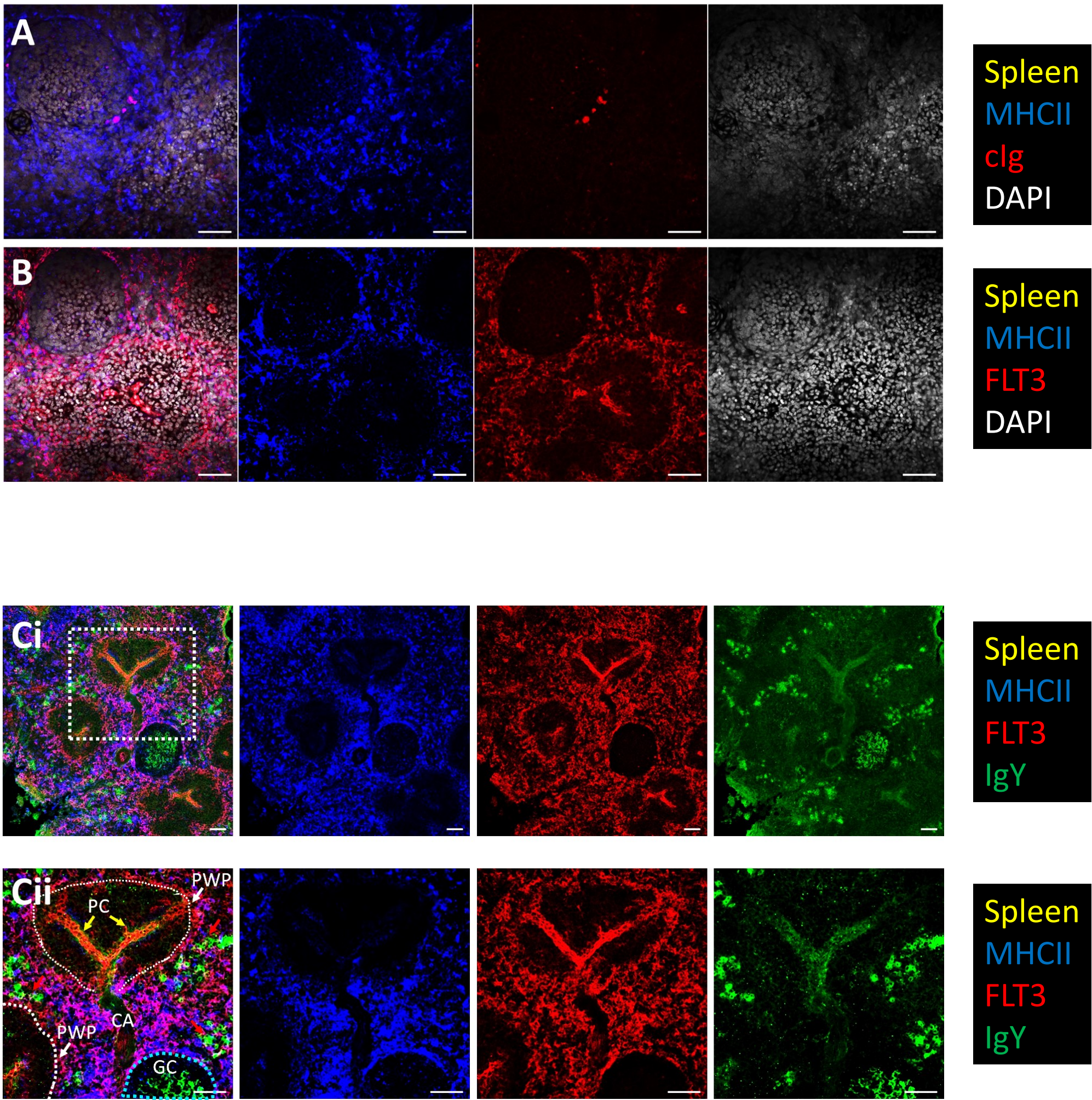

Supplement: Supplementary file 3 — Fig S3 [file IMM-165-171-s002.pdf]

Supplementary Figure 4

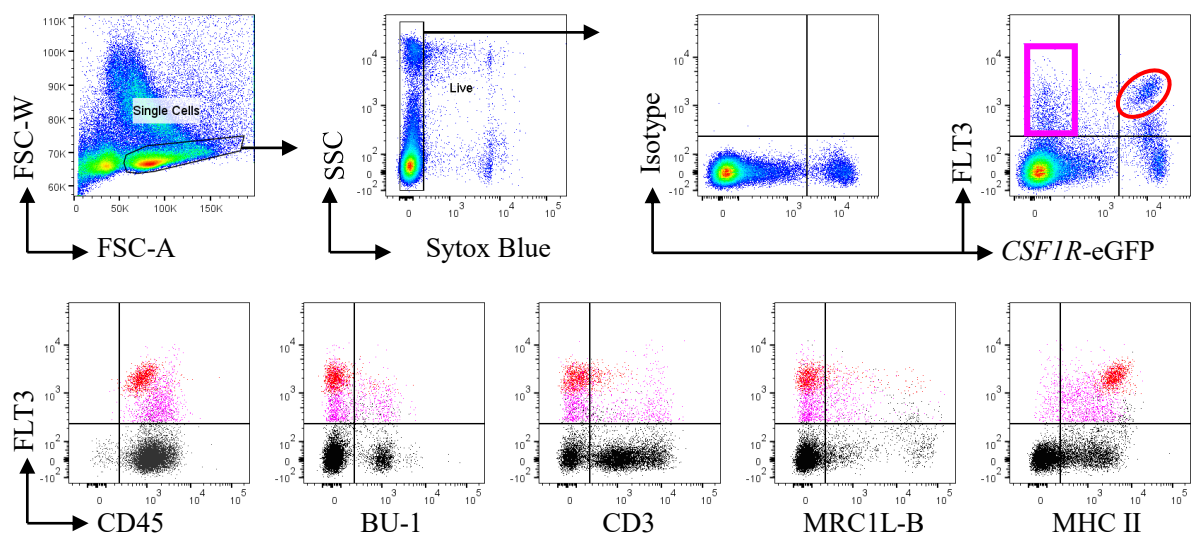

Supplement: Supplementary file 4 — Fig S4 [file IMM-165-171-s001.pdf]
